# The SIRT1 activator SRT2104 exerts exercise mimetic effects and promotes Duchenne muscular dystrophy recovery

**DOI:** 10.1038/s41419-025-07595-z

**Published:** 2025-04-07

**Authors:** Matteo Giovarelli, Silvia Zecchini, Silvia Rosanna Casati, Laura Lociuro, Oriola Gjana, Luca Mollica, Elena Pisanu, Harcel Djaya Mbissam, Ornella Cappellari, Chiara De Santis, Alessandro Arcari, Anne Bigot, Giuditta Clerici, Elisabetta Catalani, Simona Del Quondam, Annapaola Andolfo, Clarissa Braccia, Maria Grazia Cattaneo, Cristina Banfi, Dario Brunetti, Emanuele Mocciaro, Annamaria De Luca, Emilio Clementi, Davide Cervia, Cristiana Perrotta, Clara De Palma

**Affiliations:** 1 Department of Biomedical and Clinical Sciences (DIBIC), Università degli Studi di Milano, Milan, Italy; 2 Department of Medical Biotechnology and Translational Medicine (BioMeTra), Università degli Studi di Milano; Segrate, Milan, Italy; 3 Department of Pharmacy - Drug Sciences, University of Bari Aldo Moro, Bari, Italy; 4 Sorbonne Université, Inserm, Institut de Myologie, Centre de Recherche en Myologie, Paris, France; 5 Human Technopole, Milan, Italy; 6 Department for Innovation in Biological, Agro-Food and Forest Systems (DIBAF), Università degli Studi della Tuscia, Viterbo, Italy; 7 ProMeFa, Proteomics and Metabolomics Facility, Center for Omics Sciences, IRCCS San Raffaele Scientific Institute, Milan, Italy; 8 Unit of Functional Proteomics, Metabolomics and Network Analysis, Centro Cardiologico Monzino IRCCS, Milan, Italy; 9 Unità di Genetica Medica e Neurogenetica, Fondazione IRCCS Istituto Neurologico Carlo Besta, Milano, Italy; 10 Dipartimento di Scienze Cliniche e di Comunità, Dipartimento di Eccellenza 2023-2027, Università degli Studi di Milano, Milan, Italy

**Keywords:** Neuromuscular disease, Preclinical research

## Abstract

Duchenne muscular dystrophy (DMD) is a devastating genetic disorder, whose management is still a major challenge, despite progress in genetic and pharmacological disease-modifying treatments have been made. Mitochondrial dysfunctions contribute to DMD, however, there are no effective mitochondrial therapies for DMD. SIRT1 is a NAD^+^-dependent deacetylase that controls several key processes and whose impairment is involved in determining mitochondrial dysfunction in DMD. In addition to well-known resveratrol, other potent selective activators of SIRT1 exist, with better pharmacokinetics properties and a safer profile. Among these, SRT2104 is the most promising and advanced in clinical studies. Here we unveil the beneficial effects of SRT2104 in flies, mice, and patient-derived myoblasts as different models of DMD, demonstrating an anti-inflammatory, anti-fibrotic, and pro-regenerative action of the drug. We elucidate, by molecular dynamics simulations, that a conformational selection mechanism is responsible for the activation of SIRT1. Further, the impact of SRT2104 in reshaping muscle proteome and acetylome profiles has been investigated, highlighting effects that mimic those induced by exercise. Overall, our data suggest SRT2104 as a possible therapeutic candidate to successfully counteract DMD progression.

## Introduction

Duchenne Muscular Dystrophy (DMD) is an X-linked degenerative rare genetic disease caused by mutations of the *DMD* gene, encoding for dystrophin protein. DMD’s incidence is around 1 in 5000 live males and is characterized by progressive muscle weakness which untimely leads to death due to heart or respiratory failure [1, 2]. Loss of dystrophin disrupts the dystrophin-associated protein complex beneath the sarcolemma causing muscle membrane fragility and eventually leading to deleterious inflammation, fibrosis, and regenerative impairment [3]. Corticosteroids, the current standard of care, delay the loss of function and muscle breakdown, although associated with severe adverse effects [4–6]. The main goal of current clinical research is to develop new treatments that extend the lifespan and enhance the quality of life for patients with DMD.

Emerging findings suggest that mitochondrial dysfunctions are early defects in DMD muscle eliciting poor myofiber repair and contributing to fuel muscle degeneration and necrosis. In dystrophic fibers, defects in mitochondrial de novo biogenesis and mitophagic turnover affect organelles’ structure, quality, and bioenergetics [7–10]. Preserving mitochondrial homeostasis can, hence, delay skeletal muscle degeneration promoting muscular functionality.

Silent information regulator 1 (SIRT1), a NADH-dependent class III histone deacetylase of the Sirtuin family, is a stress sensor and metabolic regulator whose activity impinges on cellular energy balance and controls aging and longevity in mammals [11, 12]. As documented in the literature, the free (apo) inactive form of SIRT1 adopts a wide-open conformation characterized by the formation of a wide interdomain cleft in which the smaller N-terminal domain (NTD) and the larger catalytic domain (CD) are freely rotating in solution. Conversely, the activated form of SIRT1 displays compactness with a pocket formed to accommodate the activator molecule [13]. The physiological relevance of SIRT1 relies on the deacetylation of over 70 histone and non-histone substrates involved in gene expression regulation [14]. Increased energy demand (e.g., fasting, calorie restriction, and exercise) activates SIRT1 in skeletal muscle fostering mitochondrial biogenesis and fatty acid oxidation (FAO) and promoting anti-inflammatory activity [15–17]. Many SIRT1-activating compounds exist either as natural products (e.g., resveratrol) or selective synthetic molecules [18, 19]. Although effective in mitigating muscle injury in dystrophic mice, resveratrol is a neither potent nor selective SIRT1 activator since it acts on many biomolecules and shows limited bioavailability due to very rapid metabolism and elimination [20–26]. Among selective synthetic compounds, the SRT2104 molecule is the most advanced in clinical studies [27]. SRT2104 showed a safe profile in Phase I trials in healthy volunteers [28] and has drawn attention as potential pharmacological support for a wide range of human illnesses such as neurodegenerative diseases [29, 30], diabetic complications [31], psoriasis [32], ulcerative colitis [33], and depressive-like behaviors [34]. SRT2104 supplementation extended the lifespan in a mouse model of muscle atrophy improving motor performance, reducing inflammation, and preserving both muscle mass and mitochondrial activity [35, 36]. Of note, SRT2104 has strong anti-inflammatory activity documented in both mice [36] and humans [37] potentially ascribed to the modulation of tissue macrophages and extravascular leukocytes that reduces the release of cytokines and curbs the coagulation process. So far, SRT2104 has never been tested in muscular diseases nor muscular effects have been investigated in depth. Here, we unveil the beneficial effects of SRT2104 in fly, mouse, and human models of DMD, also exploring the SRT2104-dependent reshaping of proteome and acetylome landscapes in dystrophic muscle and its effects on metabolic processes such as FAO, ATP biosynthesis, and glycolysis. Moreover, we uncover the exercise mimetic properties of SRT2104 that, therefore, can be an attractive candidate for DMD treatment with positive effects on mitochondrial metabolism, myofiber regeneration, and muscle performance.

## Materials and methods

### Mice and treatment

All the procedures in mice conformed to the Italian law on animal care (D.L. 26/2014), as well as the European Directive (2010/63/UE), and animal experimentation was approved by the Italian Ministry of Health (approval no. 761/2022-PR). We used our colony of dystrophic mice generated crossing *mdx* mice (C57BL/10ScSnDmdmdx/J) with PhAM mice (C57BL6/129SV) and expressing a mitochondrial-specific version of Dendra2 (photo-switchable monomeric fluorescent protein, PhAM) [10]. This new dystrophic line retained all the typical features of *mdx* mice, regarding muscle performance and damage [10] and allowed us to better analyze different aspects of mitochondria physiology. As controls, we used animals with identical genetic background, generated crossing C57BL/10ScSnJ with PhAM mice. Mice were housed in an environmentally controlled room (23 ± 1 °C, 50 ± 5% humidity) with a 12-h light/dark cycle and provided food and water ad libitum. 8 week-old male *mdx* mice were treated with the SRT2104 molecule supplemented in the diet (4RF25) for 12 weeks, at the concentration of 1.33 g/kg of chow, to daily administer approximately 100 mg/kg body weight. A group of male age-matched *mdx* mice was fed with 4RF25 normal diet. Mice were randomly assigned to experimental groups. For all the experimental groups, we calculated the number of mice using Gpower software considering a power of 0.8, an alpha error of 0.05, and a variability of 20% within each group.

### Functional tests

The whole-body tension (WBT) force test was used to determine the ability of mice to exert tension in a forward-pulling maneuver that is elicited by stroking the tail of the mice [38]. It is thought to reflect the maximal acute phasic force the mouse can achieve to escape a potentially harmful event. The tails were connected to an MP150 System transducer (BIOPAC Systems, Inc. Goleta, CA, USA), and forward pulling movements were provoked by a standardized stroke of the tail with serrated forceps, and the corresponding pulling tensions were recorded using the AcqKnowledge software recording system (BIOPAC Systems, Inc. Goleta, CA, USA). Between 20 and 30 strokes of pulling tensions were generally recorded. The WBT was calculated as the average of the top ten or top five performances (WBT 5/WBT 10) normalized on the body weight of mice in grams and represents the maximum phasic tension that can be developed. To assess the WBT trend, the force test was performed before, in the middle, and at the end of the SRT2104 treatment.

In the treadmill exhaustion test, animals were trained to run on the standard treadmill machine Exer 3/6 Treadmill (Columbus Instruments, Columbus, OH, USA), according to TREAT-NMD SOP DMD_M.2.1.003 (https://www.treat-nmd.org/wp-content/uploads/2023/07/MDX-DMD_M.2.1.003.pdf).

The test consisted of horizontal running for 5 min at 8 cm/s, then the speed was increased by 2 cm/s each minute until reaching either 50 cm/s or mice exhaustion as reported in the SOP DMD_M.2.1.003. Exhaustion was defined as the inability of the animal to return to running within 10 s after direct contact with an electric stimulus grid. Running time and distance were provided by the software, while distance was calculated from time and speed. Mice were sacrificed at least 24 h after the exhaustion treadmill test.

### D. melanogaster stocks, husbandry, and treatment

The dystrophic mutant strain used in this study was Dys^E17^ [39], Oregon-R was used as the WT strain. Stocks #63047 (Dys^E17^), and #5 (Oregon-R) were obtained from Bloomington Drosophila Stock Center (Indiana University Bloomington, IN, USA). Dys^E17^ is characterized by a point mutation on chromosome 3, 92A10, 3 R:19,590,458. 19,590,458, causing a nucleotide change C19590458T and consequently the amino acid change Q2807term|Dys-PA. Third chromosome alleles were balanced with the TM6, Tb balancer chromosome. Experiments were performed with groups of 15–20 flies of the same age per vial. Flies were randomly assigned to experimental groups and raised on a standard corn meal agar food at 25 °C ± 1 °C, with controlled 12 h/12 h light/dark cycles. Adult Drosophila (3–5 days old) were then transferred in the vials containing a final volume of 10 ml of the standard diet for 21 days. SRT2104 was mixed to cool down food into vials to obtain the final working concentration ranging from 10 to 50 µg/ml. The diet containing the vehicle was used as a control diet. Diets were renewed weekly by transferring flies to a new vial containing a fresh diet.

### D. melanogaster survival and climbing

Fly survivor was documented recording the number of dead individuals per vial every 5 days, starting at day 10 of treatment. Geotaxis was assessed every 5 days from treatment using a climbing assay as previously published [39]. We generated cohorts (empty vials) consisting of ca. 20 flies. A horizontal line was drawn 15 cm above the bottom of the vial. After a 10-min rest period, the flies were tapped to the bottom of the vials: all flies were forced to start climbing (vertical walking). The number of flies that climbed up to the 15 cm mark after 60 s was recorded as the percentage success rate. A camera was recording fly movement during the experiment. Each trial was performed three times, at 1-minute intervals, and the results were averaged.

### Cell cultures

Muscle stem cells (MuSCs) were prepared from muscles following a standardized, automated tissue dissociation protocol with a gentleMACS™ Octo Dissociator with Heaters (Miltenyi Biotec, Bergisch Gladbach, Germany) and magnetic depletion as previously reported [40]. To generate myotubes, MuSCs were cultured on Matrigel-coated (BD Biosciences, San Jose, CA, USA) Ibidi µ-Slide 8 Well (Cat.No:80826, Ibidi GMBH, Gräfelfing, Germany) in DMEM (EuroClone, Pero, Milan) supplemented with 20% fetal bovine serum (EuroClone, Pero, Milan), 3% chick embryo extract (custom made), 10 ng/ml basic fibroblast growth factor (PeproTech, London, UK), 1% glutaMax (Gibco; ThermoFisher Scientific, Waltham, MA USA), and 1% penicillin-streptomycin (EuroClone, Pero, Milan). After 48 h of proliferation, cells were cultured for other 2 days in 2% horse serum (EuroClone, Pero, Milan) to induce differentiation and then harvested for the morphometric analysis of myotubes. SRT2104 treatment (3 μM in DMSO) was performed from the beginning.

Three pediatric patient-derived immortalized cell lines (AB1098 dystrophic myoblasts from a 14 years old patient with 48–50 exons deletion mutation; AB1023 dystrophic myoblasts from an 11 years old patient with a nonsense mutation in exon 59; AB1190 myoblasts isolated from a 16 years old male control), granted from the MyoLine biobank, were exploited [41]. These samples have been used according to the MTA with Prof. Mouly, director of MyoLine biobank, in the frame of the agreement with Prof. Cappellari and Prof. Giovarelli at the University of Bari and Milan, respectively. The cells were maintained in a proliferative state using basal medium (Skeletal muscle cell growth medium; PromoCell, Heidelberg, Baden-Württemberg, Germany), supplemented with 15% FBS (Gibco; ThermoFisher Scientific, Waltham, MA USA), 1% gentamicin (Gibco; ThermoFisher Scientific, Waltham, MA USA), 1% glutaMax (Gibco; ThermoFisher Scientific, Waltham, MA USA), and 5% growth medium supplement mix (PromoCell, Heidelberg, Baden-Württemberg, Germany). For myotubes formation, confluent cells were cultured in basal medium (Skeletal muscle differentiation medium; PromoCell, Heidelberg, Baden-Württemberg, Germany), supplemented with 2% FBS (Gibco, ThermoFisher Scientific, Waltham, MA USA) and 2% differentiation medium Supplemental mix (PromoCell, Heidelberg, Baden-Württemberg, Germany). SRT2104 treatment (3 μM in DMSO) was performed from the beginning and cells were harvested and analyzed after 72 h of differentiation.

For mitochondrial membrane potential analysis, 10 μl of 100 μM TMRM (MitoProbe™ TMRM Assay M20036, ThermoFisher Scientific, Waltham, MA USA) stock solution was added to 10 mL of cell growth medium to prepare the staining solution. The growth medium was removed, and the staining solution was added to the human myotubes after 72 h of SRT2104 treatment (3 μM in DMSO) in the differentiation medium. Human myotubes were then incubated for 30 min at 37 °C and washed once with PBS. Detached single cells were counted and resuspended at the concentration of 1 × 10^6^ cells/mL in PBS and the acquisition was performed on a CytoFLEX™ flow cytometer system equipped with CytExpert software (Beckman Coulter, Brea CA, USA). Data were analyzed using Kaluza software, version 2.1.1. (Beckman Coulter, Brea CA, USA). The analysis includes experiments of both hDMD1 and hDMD2 myoblasts.

MuSCs and human DMD myoblasts experiments were performed at least in experimental triplicate.

### Histology and immunofluorescence

Dissected muscles were immediately frozen to allow the preparation of 7 µm thick sections by Leica CM1860 UV cryostat (Leica Biosystems, Milan, Italy) for both morphological and immunofluorescence analysis. Hematoxylin and Eosin (H&E) staining (Bio Optica, Milan, Italy) was performed as previously described [10]. Sirius Red staining was used to quantify fibrosis: sections were fixed in 4% formaldehyde (Merck, Darmstadt, Germany), washed in distilled water, and ethanol 100% (5’). Once dried, sections were stained with 0.3% Sirius Direct Red 80 solution (Merck, Darmstadt, Germany) for 1 h RT, rinsed in distilled water, dipped twice in 0.5% acetic acid (5’), 100% EtOH (5’), cleared in Xylene (20’), and mounted. For immunofluorescence, sections were fixed with 4% paraformaldehyde for 10 min, blocked for 1 h with 5% goat serum 0,1% triton-PBS, and then incubated with primary antibodies diluted in blocking solution O/N [42]. After incubation with the appropriate fluorescent-labeled secondary antibodies, nuclei were counterstained with DAPI (1:1000 for 10 min) and finally, slides were mounted with Fluoroshield mounting medium (Merck, Darmstadt, Germany). For the evaluation of sarcolemma integrity, after fixation and blocking, sections were directly incubated with the anti-mouse IgG secondary antibody conjugated to Alexa Fluor for 1 h, washed in PBS two times, counterstained with DAPI, and mounted. Images were acquired using a Leica TCS SP8 AOBS and Stellaris microscope system, using a 40X/1.30 oil immersion objective (Leica Microsystems, Wetzlar, Germany).

For MuSCs and human myotubes immunofluorescence, cells were fixed with 4% paraformaldehyde for 10 min and permeabilized with 0.1% TritonX-100 in PBS for 5 min, then blocked for 1 h in a buffer containing 5% normal goat serum and PBS. Primary antibodies were diluted in blocking buffer and incubated overnight at 4 °C. Stained cells were washed three times with PBS and incubated with Alexa-conjugates secondary antibodies for 1 h at room temperature and nuclei were counterstained with DAPI (1:1000). Slides were mounted with Fluoreshield Mounting medium.

D. melanogaster thoraxes were immersion-fixed overnight in 4% paraformaldehyde in 0.1 M PBS at 4 °C and then transferred to 20% sucrose in PBS at 4 °C for at least 24 h. Longitudinal sections (16 μm) were obtained by a cryostat, mounted onto positively charged slides, and stored at −25 °C until use. Fluorescent phalloidin (F-actin staining, 1:1500) was used to mark muscle fibers. Images were acquired by an LSM 710 confocal microscope (Carl Zeiss, Oberkochen, Germany), with the distance between adjacent focal planes (z-stacks) set at 1 μm, and then analyzed using Adobe Photoshop (Adobe Systems, Mountain View, CA, USA) to evaluate impaired tissue areas.

The antibodies used are reported in Supplementary Table 2.

### Transmission electron microscopy

D. melanogaster thorax samples were processed for TEM in agreement with previous studies [39]. Briefly, fixed and dehydrated tissues were exposed to two steps in pure propylene oxide for 10 min each, at 4 °C. Samples were then infiltrated with mixtures of Agar 100 resin/propylene oxide in different percentages. Then, samples were embedded in pure Agar 100 resin and let to polymerize for 2 days at 60 °C. The obtained resin blocks were cut with Reichert Ultracut ultramicrotome using a diamond knife. Ultrathin sections (60–80 nm) (Leica Microsystems, Wetzlar, Germany) were collected on copper grids, stained with uranyl acetate and lead citrate, and observed with a JEOL 1200 EXII electron microscope (Jeol, Tokyo, Japan). Images were captured by the Olympus SIS VELETA CCD camera equipped with iTEM software (Olympus, Tokyo, Japan).

### SIRT1 activity assay

The deacetylase activity of SIRT1 was determined using a SIRT1 Activity Assay Kit (Abcam, Cambridge, UK) according to the manufacturer. Briefly, crude nuclear extracts from diaphragm (DP) muscles were incubated with SIRT1 assay buffer, Fluoro-Substrate peptide, and NAD^+^ respectively to initiate the reaction. The fluorescence intensity was measured for 30 to 60 minutes at 1–2 min intervals at Ex/Em = 350/460 nm. Protein was determined using a BCA protein assay kit (Thermo Scientific, Waltham, MA USA). SIRT1 activity was normalized to their respective protein concentrations and expressed as the fold change compared to the vehicle group.

### Statistical evaluation

Investigators were blinded in experimental group allocation and analysis, as well as for cell culture experiment measurements. Data were pooled from different experiments. D’Agostino and Pearson’s omnibus test was applied to assess the normal distribution of data. A comparison of two groups normally distributed was performed using an unpaired two-tailed t-test. Comparison of multiple groups was performed by one-way ANOVA followed by post hoc Tukey’s test. For grouped analyses, two-way ANOVA with correction for multiple comparisons using the Holm–Sidak method was used, while for histograms reporting different genes, we applied multiple t-tests. The GraphPad Prism software package (Graph Software) was used. The results are expressed as means ± SEM of the indicated n values. A *P* < 0.05 was considered significant. Data was excluded only when either technical error could be identified or if outliers were identified by GraphPad Prism software. Overall, no data were excluded from this study.

## Results

### SRT2104 rescued muscle performance and structure in dystrophic Drosophila

Drosophila dys gene encodes three large-isoforms of dystrophin-like protein (DLP1, DLP2, and DLP3) and three truncated products (Dp186, Dp205, and Dp117) [43]. Since the Dys gene is evolutionarily well conserved, we used a genetic loss-of-function homozygous mutant for dys, i.e., Dys^E17^, as a fly model of DMD [39, 44] to preliminarily evaluate SRT2104 effects in vivo.

**Fig. 1:**
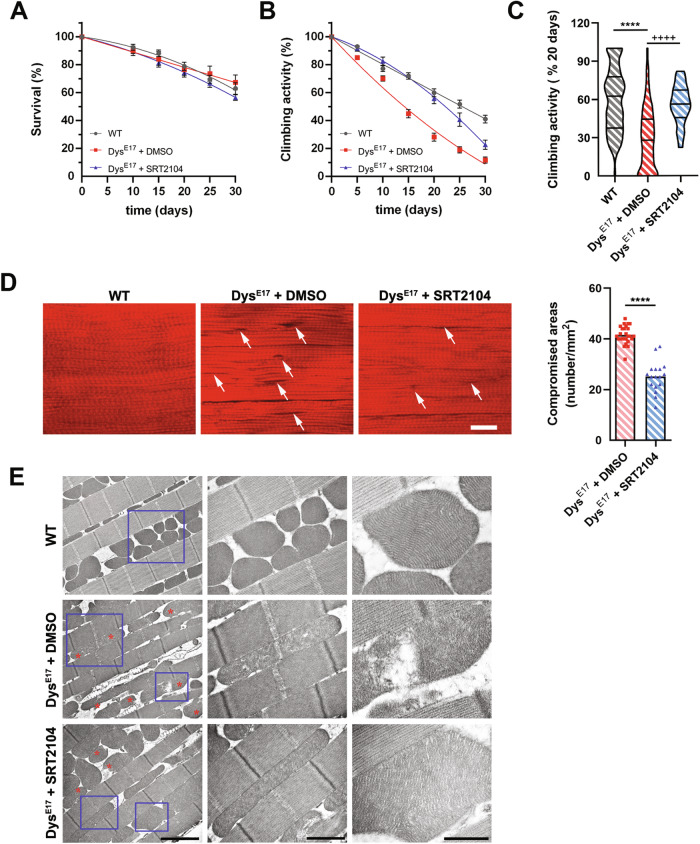
SRT2104 administration to dystrophic flies improves muscle functionality and mitochondrial morphology. **A** SRT2104 administration to dystrophic (Dys^E17^) D. melanogaster does not affect the survival rate compared to both WT and DMSO-treated flies. **B** 30 days of SRT2104 administration improves muscular performances in dystrophic flies measured by climbing activity. **C** Climbing activity measurement after 20 days of treatment is provided (right panel). * versus WT flies (**** *P* < 0.0001), + versus Dys^E17^ DMSO flies (++++ *P* < 0.0001) (WT *n* = 84; Dys^E17^ + DMSO *n* = 78; Dys^E17^ + SRT2104 *n* = 18). **D** Representative phalloidin immunostaining of WT and Dys^E17^ flies treated with either DMSO or SRT2104 (scale bar = 20 μm). Damaged fiber areas are indicated (arrows). Muscle-compromised areas for Dys^E17^ flies SRT2104 treated or not are provided (right panel) (Dys^E17^ + DMSO *n* = 26; Dys^E17^ + SRT2104 *n* = 17). * versus Dys^E17^ DMSO flies (**** *P* < 0.0001). **E** Electron microscopy analysis of WT, Dys^E17^, and Dys^E17^ + SRT2104 D. melanogaster thorax muscles. Representative insets showing mitochondrial morphologies (red asterisks) are provided (scale bar = 2 μm). Values are expressed as mean ± SEM.

SRT2104 was added to the food at 10 µM for 30 days, after which we evaluated the general behavior of adult Dys^E17^ flies and their survival rate. As shown in Fig. 1A, SRT2104 had no toxic effects when compared with untreated Dys^E17^ and the survival rate was comparable to that of the wild-type (WT) flies.

To test whether SRT2104 was effective in recovering muscle function in dystrophic drosophila, we analyzed the mobility of flies by measuring their climbing capability as a percentage of success rate. In agreement with previous reports [44, 45], the climbing decay, expressed as T_1/2_ mobility, was faster in Dys^E17^ mutants compared to WT (14.1 *vs* 25.6 days, respectively) (Fig. 1B). Of note, flies equally climb just after eclosion and the functional deficit in Dys^E17^ mutants appears later with time, thus highlighting Dys^E17^ as a model of age-dependent progression of muscular dystrophy. T_1/2_ mobility was attenuated in the presence of 10 µM SRT2104 (22.2 days) and, accordingly, when measured at 20 days, the climbing ability of Dys^E17^ was at least 53% lower compared to that of WT and it was significantly increased in SRT2104-treated groups (Fig. 1C).

We also examined the morphology of Dorsal Longitudinal Flight Muscles (DLM) at 20 days. Fluorescent phalloidin staining to visualize actin striations in longitudinal sections showed numerous non-homogeneous and degenerated areas in Dys^E17^ muscle (Fig. 1D). Fibers lost their normal linear pattern, and abnormal spaces were frequently detected between them. Dys^E17^ treatment with 10 µM SRT2104 partially restored muscle structure/pattern and significantly reduced the number of compromised areas (Fig. 1D).

Moreover, SRT2104 exerted a beneficial effect on mitochondria. Consistently, TEM images revealed disorganized cristae and damaged areas in the mitochondria of Dys^E17^ flies, while SRT2104 administration for 20 days improved their structure. Notably, the ultrastructure analysis of Dys^E17^ DLM sections confirmed muscle degeneration that was attenuated by SRT2104 treatment (Fig. 1E).

### SRT2104 rescued muscle performance and phenotypes in *mdx* mice

To test whether SRT2104 also improves the dystrophic phenotype in a mammalian DMD model, we treated 8-week-old *mdx* mice with the SRT2104 supplemented in the diet for 12 weeks, at the concentration of 1.33 g/kg of chow, to administer approximately 100 mg/kg of body weight daily [36]. The same diet without any drug was used as a control chow. We started the treatment during the muscle regeneration phase, after the acute muscle damage, to prevent the progression toward subsequent hypertrophic and fibrotic phases. Mice were weighed every week and no difference in body weight was ever detected (Supplementary Fig. 1A). Similarly, food intake was superimposable between control and treated *mdx* mice (Supplementary Fig. 1B) and no signs of toxicity appeared during the treatment confirming the safety of the drug.

**Fig. 2:**
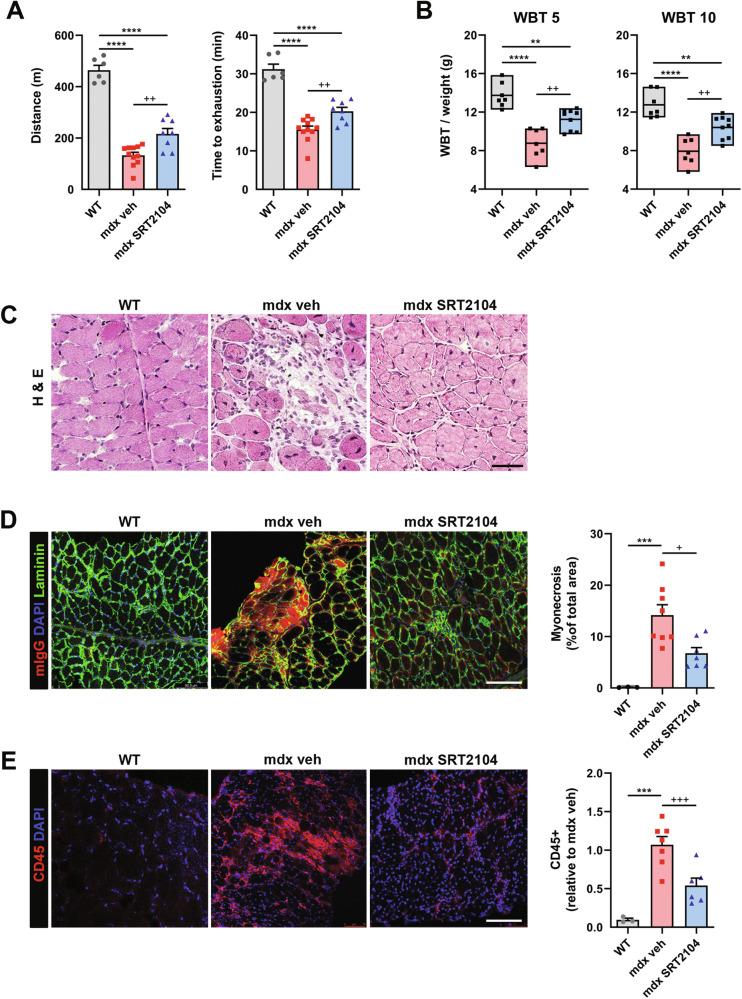
SRT2104 administration to *mdx* mice enhances muscle performance and reduces muscle damage. SRT2104 administration in *mdx* mice improves muscular performances, assessed by exhaustion treadmill running test, measuring distance ran (**A** left panel) and time to exhaustion (**A** right panel), as well as force, assessed by WBT test, with WBT 5 (**B** left panel) and 10 (**B** right panel), representing respectively the best 5 or the best 10 forward pulling tension normalized on body weight (WT *n* ≥ 6; mdx veh *n* ≥ 7; mdx SRT2104 *n* ≥ 8). Histological analyses in diaphragm (DP) muscle of WT, *mdx* vehicle, and *mdx* upon SRT2104 treatment: hematoxylin and eosin (H&E) staining (scale bar = 50 μm) (**C**); mouse IgG staining labeling myonecrotic fibers (scale bar = 100 μm) (**D**); CD45 staining to identify immune infiltrate (scale bar = 100 μm) (**E**). Percentage of myonecrosis area (WT *n* = 3; *mdx* veh *n* = 8; mdx SRT2104 *n* = 7) and CD45+ fibers relative to *mdx* vehicle (WT *n* = 3; *mdx* veh *n* = 7; *mdx* SRT2104 *n* = 6) are provided. * versus WT mice, (** *P* < 0.01, *** *P* < 0.001, **** *P* < 0.0001). + versus vehicle-treated *mdx* mice (+ *P* < 0.05, ++ *P* < 0.01, +++ *P* < 0.001). Values are expressed as mean ± SEM.

SRT2104-treated *mdx* mice performed better than those treated with a standard diet after 3 months of constant administration. Consistently, the overall capacity to sustain physical exercise has been evaluated; mice were gradually trained to run on a treadmill, and time to reach exhaustion at increased speed was measured. SRT2104-treated mice ran longer than untreated mice as witnessed by the higher distance run and the time of exhaustion (Fig. 2A). Besides, SRT2104 improved in vivo muscle force, assessed by whole-body tension (WBT) (Fig. 2B) which measures the maximal acute phasic force the mouse can achieve. The improvement was persistent up to the end of the treatment, even though 6 weeks of SRT2104 administration were sufficient to significantly increase in vivo muscle force (Supplementary Fig. 1C).

Muscle disintegrity is another peculiarity of dystrophic muscles that has been counteracted by SRT2104. Consistently, the *mdx* diaphragm (DP) was morphologically preserved upon SRT2104 exposure (Fig. 2C), with a reduced number of necrotic fibers (Fig. 2D) and invading CD45 positive inflammatory cells (Fig. 2E). The recovery was not restricted to the DP, as superimposable results were obtained in the Tibialis Anterior (TA) of *mdx* mice (Supplementary Fig. 2A, B, C).

**Fig. 3:**
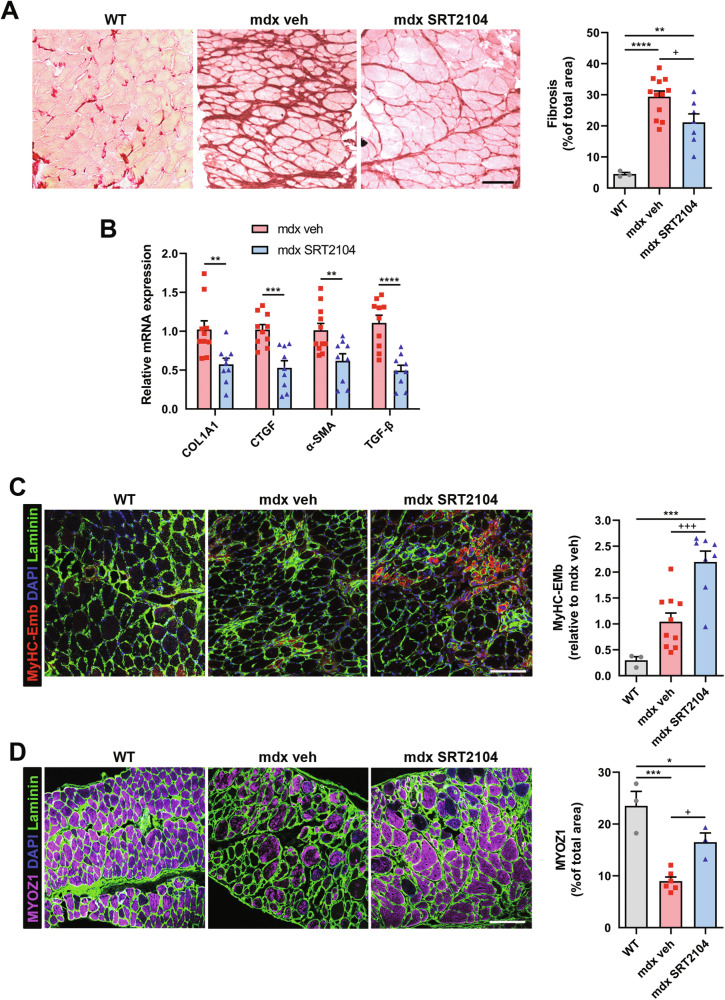
SRT2104 treatment reduces fibrosis and promotes regeneration in *mdx* mice. **A** Sirius red staining labeling fibrotic tissue in DP muscle of WT, *mdx* vehicle, and *mdx* upon SRT2104 treatment (scale bar = 100 μm). Quantification of fibrosis is provided (right panel) (WT *n* = 3; *mdx* veh *n* = 12; *mdx* SRT2104 *n* = 7). **B** RT-qPCR analysis of fibrotic genes COL1A1, CTGF, α-SMA, and TGF-β in DP of vehicle- and SRT2104-treated *mdx* mice (*mdx* veh *n* ≥ 10; *mdx* SRT2104 *n* = 9). **C**, **D** Representative immunostaining of embryonic myosin heavy chain (MyHC3-Emb) to identify regenerating fibers and MYOZ1 to assess myofibers maturity (scale bars = 100 μm). Quantification of MyHC-Emb regeneration foci relative to *mdx* vehicle (WT *n* = 3; *mdx* veh *n* = 10; *mdx* SRT2104 *n* = 8) and MYOZ1 staining on total sections area (WT *n* = 3; *mdx* veh *n* = 6; *mdx* SRT2104 *n* = 3) are provided. **A**–**D** * versus WT mice (* *P* < 0.05, ** *P* < 0.01, *** *P* < 0.001, **** *P* < 0.0001); + versus vehicle-treated *mdx* mice (+ *P* < 0.05, +++ *P* < 0.001.) **B** * versus vehicle-treated *mdx* mice (** *P* < 0.01, *** *P* < 0.001, **** *P* < 0.0001). Values are expressed as mean ± SEM.

The ability of SRT2104 treatment to counteract the DP degeneration was confirmed by a decreased collagen content in the muscle (Fig. 3A), as well as reduced expression of fibrotic and adipogenic genes (Fig. 3B and Supplementary Fig. 2D).

In parallel, DP muscle regeneration was improved as indicated by the increased number of regenerating fibers positive for embryonic myosin heavy chain (eMyHC) (Fig. 3C). Since eMyHC labeled only immature regenerating myofibers, we assessed the extent of muscle regeneration by evaluating the expression of the sarcomere protein myozenin (MYOZ1) as a reliable marker of myofiber maturity [46]. As expected, the number of MYOZ1-positive fibers increased after SRT2104 treatment (Fig. 3D), suggesting a role for SRT2104 in preventing muscle degeneration by promoting complete muscle regeneration.

Likewise, SRT2104 reduced muscle senescence (Supplementary Fig. 3A) and increased the myogenic potential of dystrophic MuSC-derived myoblasts. MuSCs were sorted from 2-month-old *mdx* muscles [40, 47, 48], activated into myoblasts in proliferation conditions for two days, and then switched in differentiation conditions to mature and fuse into myotubes in the presence or absence of SRT2104 (3 μM) from the beginning. The treatment promoted MuSCs’ ability to fuse and form myotubes (Supplementary Fig. 3B), leading to an increased number of nuclei per myotube (Supplementary Fig. 3B) and indicating that SRT2104 retained the myogenic potential of MuSCs in dystrophic conditions.

Overall, our results suggest that the ability of SRT2104 to promote muscle regeneration and less inflammation and fibrosis can explain the protective effects of the drug against muscle damage.

### SRT2104-dependent SIRT1 activation modified the protein acetylation landscape of dystrophic muscle

**Fig. 4:**
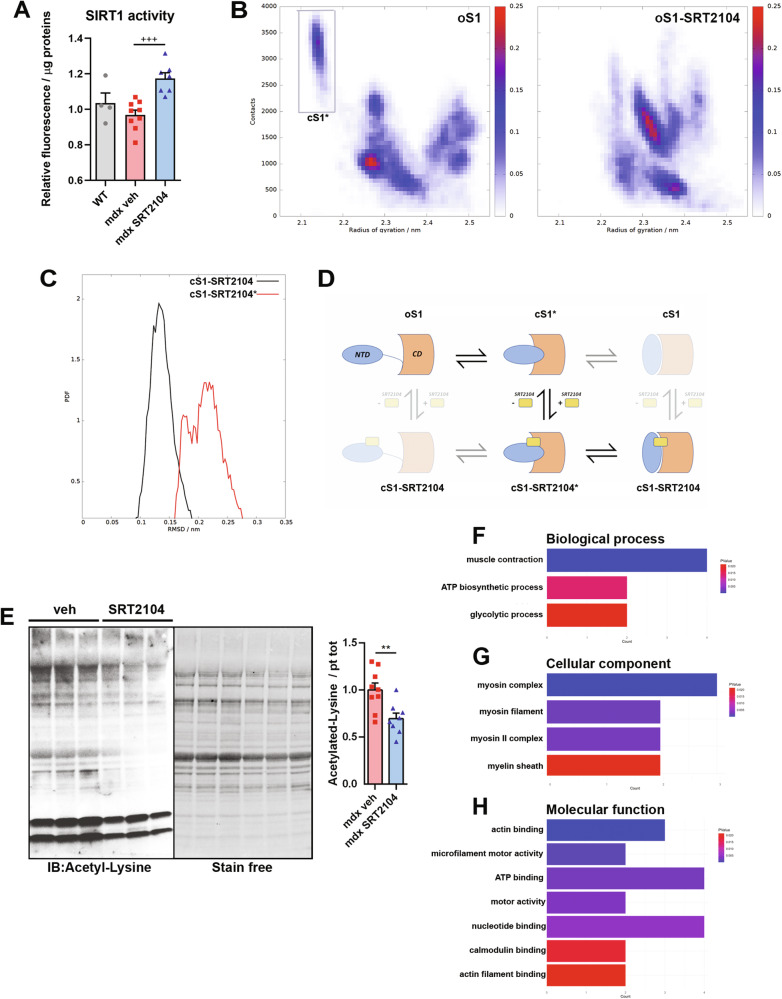
SRT2104 treatment activates SIRT1 leading to deacetylated protein pattern reshape. **A** Fluorescence-based SIRT1 activity assay using crude nuclear extracts of DP muscles, normalized to protein content (WT *n* = 4; *mdx* veh *n* = 9; *mdx* SRT2104 *n* = 7). + versus vehicle-treated *mdx* mice (+++ *P* < 0.001). Values are expressed as mean ± SEM. **B** Maps of the populations of the three-dimensional configurations explored by the open apo form of SIRT1 (left, oS1) and by the open holo form of SIRT1 (right, oS1-SRT2104) through molecular dynamics (MD) simulations. The geometric states are reported in the form of correlation maps between the intermolecular number of contacts and the overall radius of gyration. The populations have been normalized to 1 corresponding to the highest population value for each distribution. The family of structures (cS1*) characterized by more compactness and a high number of contacts have been highlighted by a gray rectangle. **C** Distribution of the backbone heavy atoms (Ca, C’, N) root mean square deviations (RMSDs) of the holo form of two closed configurations of SIRT1, i.e., the one obtained docking with SRT2014 the active structure reported in the literature (black line: cS1-SRT2104, adapted from the PDB ID 5zzh) and the one obtained docking the compact configuration obtained simulating the dynamics of the apo form of the inactive/open conformation (red line: cS1-SRT2104*). **D** Scheme of all the possible interconversion mechanisms between the open/inactive form and the closed/active form of SIRT1 upon SRT2104 binding (NTD: N terminal domain; CD: catalytic domain): the apo forms of the open and closed conformations of SIRT1 (oS1, cS1); the holo forms of the open and closed conformations of SIRT1 complexed with SRT2104 (oS1-SRT2104, cS1-SRT2104); the apo and holo forms of the final configurations after MD simulations of the open conformation of SIRT1 (cS1*, cS1-SRT2104*). The configurations that do not directly enter the activation mechanisms are shaded. **E** Representative immunoblot using Acetyl-lysine antibody to detect lysine acetylation pattern in GC muscle homogenates of *mdx* mice treated or not with SRT2104. Quantification of protein acetylation pattern is provided (right panel) (*mdx* veh *n* = 9; *mdx* SRT2104 *n* = 8). * versus vehicle-treated *mdx* mice (** *P* < 0.01). Values are expressed as mean ± SEM. **F**–**H** Gene Ontology (GO) terms enrichment analyses of deacetylated proteins after SRT2104 treatment.

SRT2104 is a synthetic activator of SIRT1 and, consistently, the enzyme activity, measured in muscle homogenates, was enhanced (Fig. 4A), while SIRT1 protein levels remained unchanged after sustained drug exposure (Supplementary Fig. 4A). Interestingly, we performed the assay by adding an established concentration of NAD^+^ excluding cofactor levels as a variable of the test. Therefore we evaluated specific alterations in the enzyme’s capability to perform the deacetylation reaction. Our results showed that with an equal amount of cofactor, SIRT1 activity did not differ between WT and *mdx* mice. By contrast, SRT2104 enhanced the enzyme’s deacetylase activity, suggesting that at constant NAD^+^ concentration, the enzyme worked better after SRT2104 treatment.

Using available experimental structural information in combination with molecular simulations, we hypothesize a physio-chemical mechanism of activation of SIRT1 upon SRT2104 binding.

To elucidate the interconversion mechanism between the open and the closed configurations of the enzyme, we first simulated, using molecular dynamics simulations (MD), apo and holo forms of open SIRT1 (oS1 and oS1-SRT, respectively) to test whether the presence of the ligand on the surface of the protein could drive it towards a closed/active conformation. The correlation between SIRT1 compactness and the internal contacts between NTD and CD (Fig. 4B) revealed the presence of a closed form of apo SIRT1 (cS1*) absent in the holo system. This result suggests the presence of a conformational selection mechanism, where the interdomain dynamics of SIRT1 generate a conformer (cS1) able to bind a ligand and evolve toward the active form [13].

To test this hypothesis, we generated via molecular docking the SIRT1 complex with SRT2104 from the configuration cS1* (cS1-SRT*), alongside the complex from the experimental structure of activated SIRT1 (cS1-SRT) [13]. Subsequent MD simulations displayed wider mobility of the cS1-SRT* complex than those of cS1-SRT measured with respect to the geometry of the experimental active structure (backbone root mean square deviation, RMSD: Fig. 4C). The larger mobility of the former ensured a partial overlap with the structures explored by the latter, supporting the hypothesis that cS1-SRT* already has the features of the active form of SIRT1, suggesting an overall activation mechanism of the enzyme (Fig. 4D) driven by SRT2104 binding.

As expected by SIRT1 activation, the pattern of bulk protein acetylation unveiled overall reduced acetylation in SRT2104-treated TA (Fig. 4E). Besides, combining an efficient method for protein extraction with lysine-acetylated peptide immunoprecipitation and high-accuracy tandem mass spectrometry measurements, we elucidated the landscape of acetylated protein of dystrophic TA after SRT2104 treatment. A total of 141 acetylated sites were identified and, of these, 28 were differentially acetylated between the two groups (Supplementary Table 3). The site-specific heatmap analysis proved a sharp boundary between the vehicle and SRT2104-treated groups (Supplementary Fig. 5A), indicating that SRT2104 promotes significant changes in the acetylation levels of several proteins. Gene ontology (GO) analysis of biological processes revealed significant protein enrichment (*p* < 0.05) in processes involved in skeletal muscle contraction, negative regulation of apoptosis, and metabolic processes such as ATP biosynthesis and glycolysis (Supplementary Fig. 5B upper panel). Cellular components analysis indicated that differentially acetylated proteins were enriched in myosin complexes and mitochondria (Supplementary Fig. 5B lower panel). This is consistent with the landscape of acetylated lysine induced by aerobic exercise [49], featuring SRT2104 as an exercise mimetic, and confirms the relevance of this post-translation modification for muscle contraction and metabolic enzymes.

Of note, according to an enhanced SIRT1 activity, 18 lysine residues were found specifically deacetylated after SRT2104 treatment, and the corresponding acetylated proteins were significantly enriched in biological processes such as muscle contraction, ATP biosynthesis, and glycolysis (Fig. 4F). The analysis of cellular components showed that SRT2104 remarkably modified the acetylation of myosin filaments and myosin complexes (Fig. 4G) mainly affecting functions such as motor activity, actin binding, and ATP binding (Fig. 4H). These findings are in line with the changes in lysine acetylation profile upon exercise endorsing the idea of SRT2104 as exercise mimetics.

### SRT2104 activated muscle metabolism and restored mitochondrial respiratory capacity in dystrophic muscle

Along with protein acetylation, long-term SIRT1 activation can affect the expression of several proteins, thus a quantitative proteomics approach was employed to identify SIRT1-dependent proteome changes in both gastrocnemius (GS) and TA after SRT2104 administration.

**Fig. 5:**
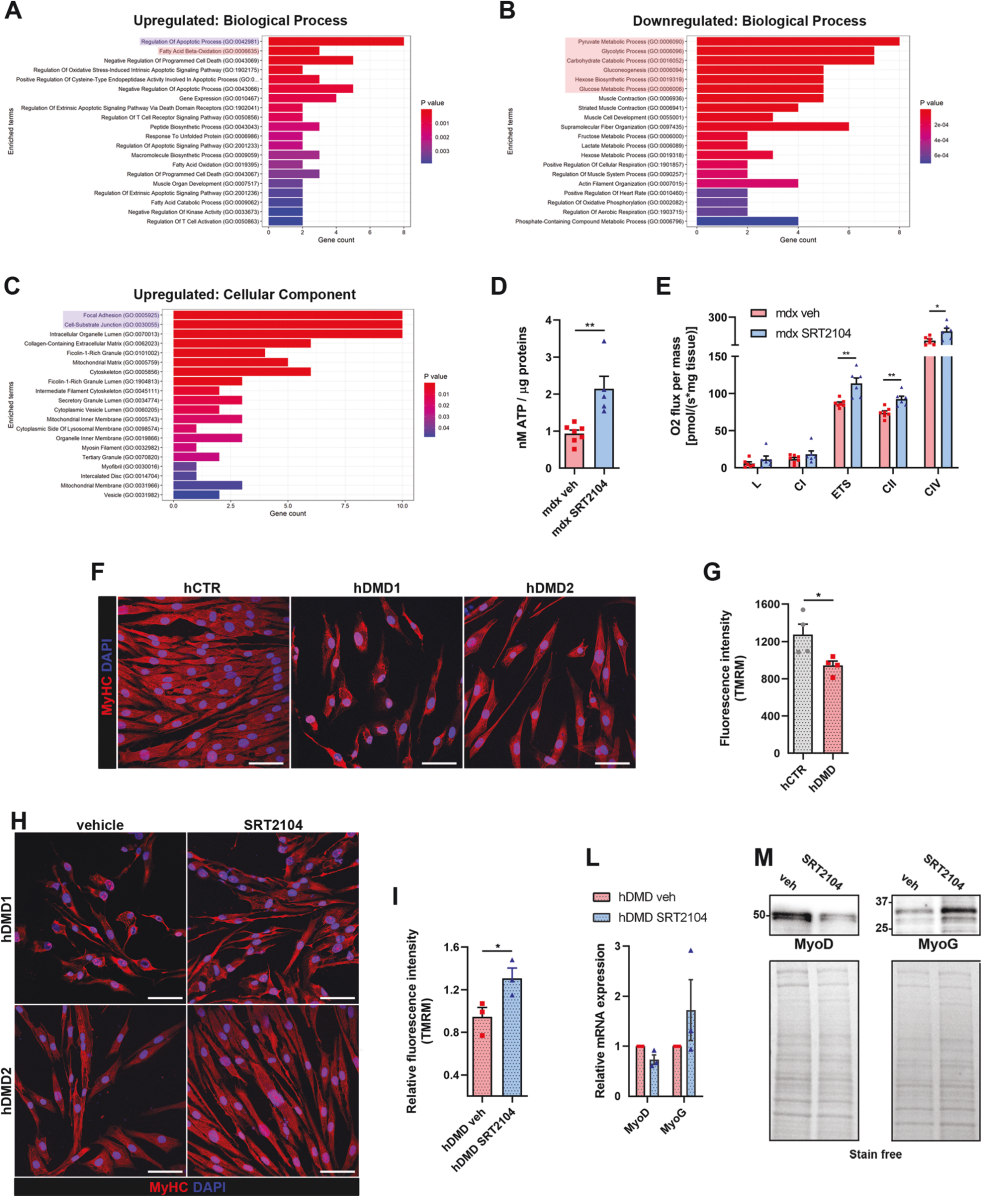
SRT2104 boosted dystrophic muscle metabolism improving mitochondrial bioenergetics. **A**–**C** GO terms enrichment analysis of differentially expressed proteins (upregulated for Biological Processes and Cellular Components; downregulated for Biological Processes) after SRT2104 treatment in GC muscle. **D** Luminescence-based ATP quantification measured in GC homogenates from *mdx* mice treated or not with SRT2104 (*mdx* veh *n* = 7; *mdx* SRT2104 *n* = 5). **E** Mitochondrial respiratory rates of permeabilized fibers’ bundles of DP muscles from *mdx* mice, treated or not with SRT2104, measured by high-resolution respirometry (HRR) (L: leak state; CI: complex I respiration; ETS: electron transfer system maximal capacity; CII: complex II respiration; CIV: complex IV activity) (*mdx* veh *n* ≥ 9; *mdx* SRT2104 *n* = 6). Representative immunostainings (red: myosin heavy chain; blue: DAPI nuclei. Scale bar = 50 μm) (**F**) and TMRM fluorescence levels (indicating basal mitochondrial membrane potential) (**G**) of human CTR and DMD myotubes in differentiation medium for 72 h (hCTR *n* = 3; hDMD *n* = 3). * versus hCTR (* *P* < 0.05). Representative immunostainings (red: myosin heavy chain; blue: DAPI nuclei. Scale bar = 50 μm) (**H**) and TMRM fluorescence levels (**I**) of human DMD myotubes treated with either DMSO (vehicle) or SRT2104 (3 μM) in differentiation medium for 72 h (hDMD veh *n* = 4; hDMD SRT2104 *n* = 4). * versus vehicle-treated hDMD (* *P* < 0.05). **L** RT-qPCR analysis of MyoD and MyoG genes in DMD patient-derived myoblasts treated or not with SRT2104 (individual values are the average of the 2 myoblast lines and the experiment has been repeated three times, *n* = 3). **M** Representative immunoblot for MyoD and MyoG proteins extracted from hDMD myoblasts treated or not with SRT2104. Molecular weights (kDa) are indicated on the left. Values are expressed as mean ± SEM.

In GS, clusters analysis showed good separation between the vehicle and SRT2104 treated groups (Supplementary Fig. 5C); among detected proteins, 74 were significantly altered following SRT2104 administration according to expression level (log_2_fold-change) and statistical significance (log_10_Padjusted). Of the differentially abundant proteins, 30 were upregulated and 44 downregulated by the treatment. To get more insight, functional enrichment analysis was performed unveiling the upregulation of FAO metabolic pathway (Fig. 5A red box) associated with the downregulation of glucose-related metabolism (Fig. 5B red box). This suggests that SRT2104 could promote the use of fatty acid over glucose, according to the key role of SIRT1 in lipolysis and FAO. Moreover, we highlighted a favorable regulation of the apoptotic process (Fig. 5A blu box) and improvements of the mechanotransduction system (Fig. 5C blue box), both supporting the observed muscle recovery. These results paralleled well with those obtained in TA in which 44 of the detected proteins were significantly modified after SRT2104 treatment with great separation between the two sample groups (*p* < 0.05 Supplementary Fig. 5D). Biological processes analysis confirmed the enrichment in metabolic processes and cytoskeleton organization, supporting favorable variations in metabolism and mechanotransduction (Supplementary Fig. 5E).

In accordance, mitochondria were metabolically more active as SRT2104 led to higher ATP levels in SRT2104-treated GS muscle (Fig. 5D). Evaluation of respiratory chain complexes activity evidenced positive effects on each respiratory complex except CI (Supplementary Fig. 6A), evidencing SRT2104 as a drug able to bypass the complex I deficiency reported in DMD [50]. Moreover, SRT2104 boosted the oxygen consumption rate of cryopreserved DP fibers’ bundles showing an increase in the maximal electron transport system (ETS) capacity fueled by complex II-driven respiration (CII) (Fig. 5E). These metabolic improvements were not coupled with an increased mitochondrial abundance since mtDNA/nuDNA ratio, as well as mitochondrial protein expression, did not differ in SRT2104-treated GS compared to vehicle (Supplementary Fig. 6B, C). Therefore, SRT2104 enhances the mitochondrial respiratory capacity of dystrophic muscle and this beneficial effect might primarily derive from beta-oxidation induction. Moreover, we tested SRT2104 efficacy in two DMD patient-derived immortalized myoblasts as a proof of concept of beneficial effects in humans. The myoblasts proliferated for two days and then were switched into the differentiation medium for 72 h, adding SRT2104 (3μM) from the beginning. As expected [51], hDMD myoblasts differentiation was impaired (Fig. 5F), as well as the mitochondrial membrane potential (MMP) (Fig. 5G), compared to hCTR line. SRT2104 treatment rescued the myogenic differentiation of hDMD myoblasts (Fig. 5H) and improved MMP (Fig. 5I), confirming its positive effects on both myogenesis and mitochondria (Fig. 5I)

Accondingly we found a reduction in MyoD expression together with increased expression of MyoG (Fig. 5L, M). The SIRT1-dependent repression of MyoD is crucial during the transition from quiescent to proliferation state downstream to metabolic reprogramming [52], while MyoG determines the terminal differentiation of myogenic progenitor cells [53, 54].

Overall, these findings, even if preliminary, almost recapitulate the data obtained in mice, confirming the therapeutic potential of SRT2104 in DMD.

## Discussion

SIRT1 belongs to a class of NAD^+^-dependent class III histone/deacetylase proteins and exerts its action by deacetylating histone and non-histone proteins.

SIRT1 is expressed in skeletal muscle and its specific deletion establishes pathological features similar to those observed in dystrophic mice [55]. By contrast, its overexpression leads to a fiber-type switch towards a slower phenotype preventing atrophy and degeneration [56]. Crossing SIRT1 transgenic mice with the dystrophic ones promotes a remarkable improvement in muscular parameters and performance [56], highlighting the crucial role of SIRT1 in DMD pathology.

Different attempts have been made to increase SIRT1 expression or activity in DMD mice and resveratrol is the pioneering compound [20, 22, 57–61]. Resveratrol is an allosteric modulator that affects K_m_ values for both NAD^+^ and the acetylated peptide, stabilizing the force between substrates and SIRT1 [62]. However, resveratrol is known to act on a wide range of enzymes and its specificity is debated as well as the effects mediated by the SIRT1 direct activation [63]. Of note, resveratrol has also a low bioavailability; after absorption, it is rapidly metabolized in the liver by glucuronidation or conjugation with sulfate [64]. The enzymes involved in the process are encoded by polymorphic genes and this results in different abilities to metabolize the drug among the population, owing to the great variability of effects [25, 26, 63, 64]. Moreover, the level of resveratrol metabolites is higher than those of the free drug, making it unclear which molecule is responsible for the observed effects [65], and its quinone metabolites also exhibit cytotoxic effects [27].

Among the alternative pharmacological options, SRT2104 is a highly selective and potent SIRT1 activator [63] since it is 1000 times more effective in activating SIRT1 deacetylase activity [27]. SRT2104 supplementation extended the lifespan of male mice; it enhanced motor coordination and performance, bone mineral density, insulin sensitivity, and reduced inflammation [36]. Of relevance, it was also found to be safe and well-tolerated in Phase I trials with healthy volunteers [28] with amelioration in plasma lipid profile, including a decrease in serum cholesterol and triglycerides [66]. SRT2104 was effective in mediating weight loss in patients with type 2 diabetes, even if it exerted inadequate control of insulin resistance [67, 68]. SRT2104 successfully crosses the blood-brain barrier [30] and exhibits promising effects in relieving depressive-like behaviors [34]. Similarly, SRT2104 promotes improvements in cognitive functions in a rat model of diabetes-induced cognitive damage, potentiating dendrites’ growth and increasing their density in the hippocampus [69].

In this study, we treated DMD flies and mice with SRT2104, constantly administered with the diet to maximize SRT2104 exposure, since it suffered from food effect and accelerated gastric emptying [28], thus repeated dose accumulation and prolonged gastric retention was a viable way to increase exposure and reduce variability [28].

We provide evidence that SRT2104 rescued dystrophic muscle damage, and its positive impact translated into improved muscle function in both models. Prolonged SRT2104 exposure of *mdx* mice reduced diaphragm fibrosis, inflammation, and senescence, and promoted regeneration. This has great translational relevance since one of the most common causes of death for patients is respiratory failure due to diaphragm impairment.

Exposure for 12 weeks is one of the longer treatments with SRT2104 to date [27] and the improvements persisted up to the end of the treatment, even if the beneficial effects could be also observed at mid-term. In parallel, the SIRT1 activation was detectable at the endpoint with clear deacetylation of the protein landscape. Of note, SIRT1 activity did not differ between WT and *mdx* mice, however, the assay does not take into account that in *mdx* mice the NAD^+^ levels are reduced as the result of increased levels of the enzymes that are the major consumers of NAD^+^ [8], as well as lower levels of the enzymes for NAD^+^ biosynthesis [8, 56]. Therefore, we can not exclude that this alteration could affect basal SIRT1 activity in dystrophic conditions. However, our result points out that at constant NAD^+^ concentration, the enzyme works better after SRT2104 treatment.

To explain the activity of the drug, by an in silico approach we proved that SRT2104 binding promoted a shift towards a closed/active conformation of SIRT1, evidencing a conformational selection mechanism of action that led to the deacetylation of substrates. Consistently, acetylome analysis revealed an interesting pattern that was superimposable with the profile of acetylated lysines upon repeated exercise [49]. The relevance of this post-translation modification (PTM) is always associated with gene regulation; however, its role extends beyond nuclear histone modifications. Protein acetylation controls sarcomere function, including myofilament contractile activity [70], and allows the optimization of energy usage [71], suggesting that lysine acetylation can impact muscle protein binding energy during exercise.

After exercise, myosin-4 (Myh4), myosin-1 (Myh1), and titin are differentially acetylated, while their protein expression does not change [49], Similarly, SRT2104 induced deacetylation of Myh4 and Myh1 (Supplementary Table 3), even if the identified sites were different. Of note, as in physiological conditions, the SRT2104-dependent deacetylation mainly occurred on Myh4 compared to Myh1, hence we can speculate that this is consistent with proper muscle performance. In this respect, we are aware that molecular simulations (i.e., docking and/or molecular dynamics) could constitute a necessary step to identify whether different lysine deacetylations might contribute to the contractile activity of the proteins.

Protein acetylation also affects the catalytic activity of metabolic enzymes [72] and, interestingly upon SRT2104 treatment glycolytic enzymes such as muscle creatine kinase (CKM) and muscle pyruvate kinase (PKM) were deacetylated (Supplementary Table 3). PKM has been identified as a SIRT1 substrate indeed SIRT1 can directly bind and deacetylate PKM increasing its activity and producing more ATP [73]. Similarly, CKM acetylation is negatively correlated with its activity, impinging the phosphotransfer reaction between phosphocreatine and ATP. Lysine 196 (K196) is one of the sites interested in the formation of homodimeric configuration required for the activity of the enzyme [74], but many other lysines have been identified whose acetylation disrupts monomer formation [49]. Accordingly, we found that the K196 and K172 sites (Supplementary Table 3) were deacetylated after SRT2104 treatment, suggesting an enhanced activity of the enzyme and likely providing an efficient energy shuttle mechanism. We conclude that the acetylome profile of SRT2104-treated muscle revealed a landscape similar to those induced by repeated exercise, with enrichment in sarcomeric protein and glycolytic enzymes [49], thus highlighting SRT2104 as an exercise mimetic contributing to efficient energy production to sustain ameliorated muscle contraction. This is relevant in DMD which shows exercise intolerance and dystrophic muscle is unable to metabolically adapt to endurance exercise for the intrinsic failure of upstream mechanical signals [75].

Noteworthy, some glycolytic enzymes were downregulated by SRT2104 treatment as reported in our proteomic analysis, however, this could be balanced by enhanced activity of other enzymes considering the relevant role of PTMs in controlling this aspect.

Another key evidence was that mitochondria were more active upon SRT2104 treatment, highlighting the role of SRT2104 in improving mitochondrial function [35]. Similarly, NAD^+^ repletion with NR results in the rescue of NAD^+^-dependent sirtuin signaling attenuating the loss of mitochondrial functionality by lowering energy stress, and reducing the susceptibility to muscle degeneration [8].

Noteworthy, SRT2104 enhanced mitochondrial activity by shunting complex I deficiency [50] and without a specific induction of mitochondrial biogenesis; conversely, it enhanced FAO, as pointed out by proteomic analysis. Accordingly, other SIRT1 activators, such as molsidomine [76] and SRT1720 [77], can promote oxidative metabolism by controlling FAO, driving a selective metabolic adaptation without modifying mitochondrial content [77]. Therefore mitochondrial abundance and respiration are not modulated at the same time, suggesting that SRT2104 improves mitochondrial function mainly acting upstream, by increasing fatty acid utilization and potentially boosting protective antioxidant mechanisms such as superoxide dismutase activity [27, 35, 36]. This also confirms that mitochondria biogenesis is impaired in DMD and requires modification of the chromatin structure at the PGC-1α promoter, by HDACi, to be effectively unlocked [10, 76]. SIRT1 activation is not the only attempt to restore mitochondrial functionality in DMD. Recently, H_2_S-releasing drugs have shown benefits in different DMD models [78–80] mainly due to improvements in mitochondrial structure and function, especially ATP synthesis and prevention of oxidant formation [81] Altogether these approaches succeed where others failed, indeed some master energy regulators are unable to induce positive adaptation of oxidative metabolism [10, 82, 83].

Last, the evidence provided in patients-derived myoblasts supports the translational potential of SRT2104, also sustained by the large amount of data collected in different clinical studies thus facilitating the process of approval for DMD.

Moreover, genetic therapies can directly correct the disease-causing mutation, however, it is still unclear whether this is enough to ameliorate secondary symptoms, such as mitochondrial dysfunction. Additionally, genetic tools need to be optimized to enhance their efficiency and, unfortunately, they can only correct one or a subset of mutations at a time. Therefore, a dual treatment based on the combination of pharmacologic and genetic tools may be recommended to improve the efficacy of DMD therapy. Hence, further studies are ongoing to elucidate the putative synergistic effects of combining SRT2104 with gene therapy to enhance the lifespan and the quality of life in patients with DMD.

## Supplementary information

Supplementary material

uncropped original western blots

## Data Availability

TA proteomic data are available via ProteomeXchange with the identifier PXD054747 and 10.6019/PXD054747. GS proteomic data are available via ProteomeXchange with identifier PXD055878 and 10.6019/PXD055878. The datasets generated and/or analyzed during the current study are available from the corresponding author upon reasonable request.

## References

[1] Emery AE. Population frequencies of inherited neuromuscular diseases-a world survey. Neuromuscul Disord. 1991;1:19–29. 1822774 10.1016/0960-8966(91)90039-u

[2] Mah JK, Korngut L, Dykeman J, Day L, Pringsheim T, Jette N. A systematic review and meta-analysis on the epidemiology of Duchenne and Becker muscular dystrophy. Neuromuscular Disord. 2014;24:482–91. 10.1016/j.nmd.2014.03.008 24780148

[3] Bez Batti Angulski A, Hosny N, Cohen H, Martin AA, Hahn D, Bauer J, et al. Duchenne muscular dystrophy: disease mechanism and therapeutic strategies. Front Physiol. 2023;14:1183101. 37435300 10.3389/fphys.2023.1183101 PMC10330733

[4] Kourakis S, Timpani CA, Campelj DG, Hafner P, Gueven N, Fischer D, et al. Standard of care versus new-wave corticosteroids in the treatment of Duchenne muscular dystrophy: can we do better? Orphanet J Rare Dis. 2021;16:117. 33663533 10.1186/s13023-021-01758-9 PMC7934375

[5] Heydemann A, Siemionow M. A brief review of Duchenne muscular dystrophy treatment options, with an emphasis on two novel strategies. Biomedicines. 2023;11:830. 10.3390/biomedicines11030830 PMC10044847 36979809

[6] Fiorillo AA, Tully CB, Damsker JM, Nagaraju K, Hoffman EP, Heier CR. Muscle miRNAome shows suppression of chronic inflammatory miRNAs with both prednisone and vamorolone. Physiol Genom. 2018;50:735–45. 10.1152/physiolgenomics.00134.2017 PMC6172612 29883261

[7] Vila MC, Rayavarapu S, Hogarth MW, Van der Meulen JH, Horn A, Defour A, et al. Mitochondria mediate cell membrane repair and contribute to Duchenne muscular dystrophy. Cell Death Differ. 2017;24:330–42. 27834955 10.1038/cdd.2016.127 PMC5299714

[8] Ryu D, Zhang H, Ropelle ER, Sorrentino V, Mazala DA, Mouchiroud L, et al. NAD+ repletion improves muscle function in muscular dystrophy and counters global PARylation. Science Transl Med. 2016;8:361ra139. 10.1126/scitranslmed.aaf5504 PMC5535761 27798264

[9] Percival JM, Siegel MP, Knowels G, Marcinek DJ. Defects in mitochondrial localization and ATP synthesis in the mdx mouse model of Duchenne muscular dystrophy are not alleviated by PDE5 inhibition. Hum Mol Genet. 2013;22:153–67. 23049075 10.1093/hmg/dds415 PMC3522404

[10] Giovarelli M, Zecchini S, Catarinella G, Moscheni C, Sartori P, Barbieri C, et al. Givinostat as metabolic enhancer reverting mitochondrial biogenesis deficit in Duchenne Muscular Dystrophy. Pharmacol Res. 2021;170:105751. 34197911 10.1016/j.phrs.2021.105751

[11] Rajendran R, Garva R, Krstic-Demonacos M, Demonacos C. Sirtuins: molecular traffic lights in the crossroad of oxidative stress, chromatin remodeling, and transcription. J Biomed Biotechnol. 2011;2011:368276. 21912480 10.1155/2011/368276 PMC3168296

[12] Michan S, Sinclair D. Sirtuins in mammals: insights into their biological function. Biochem J. 2007;404:1–13. 17447894 10.1042/BJ20070140 PMC2753453

[13] Cao D, Wang M, Qiu X, Liu D, Jiang H, Yang N, et al. Structural basis for allosteric, substrate-dependent stimulation of SIRT1 activity by resveratrol. Genes Dev. 2015;29:1316–25. 26109052 10.1101/gad.265462.115 PMC4495401

[14] Zhang T, Kraus WL. SIRT1-dependent regulation of chromatin and transcription: linking NAD(+) metabolism and signaling to the control of cellular functions. Biochim Biophys Acta. 2010;1804:1666–75. 19879981 10.1016/j.bbapap.2009.10.022 PMC2886162

[15] Canto C, Jiang LQ, Deshmukh AS, Mataki C, Coste A, Lagouge M, et al. Interdependence of AMPK and SIRT1 for metabolic adaptation to fasting and exercise in skeletal muscle. Cell Metab. 2010;11:213–9. 20197054 10.1016/j.cmet.2010.02.006 PMC3616265

[16] Gerhart-Hines Z, Rodgers JT, Bare O, Lerin C, Kim SH, Mostoslavsky R, et al. Metabolic control of muscle mitochondrial function and fatty acid oxidation through SIRT1/PGC-1alpha. EMBO J. 2007;26:1913–23. 17347648 10.1038/sj.emboj.7601633 PMC1847661

[17] Singh V, Ubaid S. Role of Silent Information Regulator 1 (SIRT1) in regulating oxidative stress and inflammation. Inflammation. 2020;43:1589–98. 32410071 10.1007/s10753-020-01242-9

[18] Hubbard BP, Gomes AP, Dai H, Li J, Case AW, Considine T, et al. Evidence for a common mechanism of SIRT1 regulation by allosteric activators. Science. 2013;339:1216–9. 23471411 10.1126/science.1231097 PMC3799917

[19] Milne JC, Lambert PD, Schenk S, Carney DP, Smith JJ, Gagne DJ, et al. Small molecule activators of SIRT1 as therapeutics for the treatment of type 2 diabetes. Nature. 2007;450:712–6. 18046409 10.1038/nature06261 PMC2753457

[20] Hori YS, Kuno A, Hosoda R, Tanno M, Miura T, Shimamoto K, et al. Resveratrol ameliorates muscular pathology in the dystrophic mdx mouse, a model for Duchenne muscular dystrophy. J Pharm Exp Ther. 2011;338:784–94. 10.1124/jpet.111.183210 21652783

[21] Kuno A, Tanno M, Horio Y. The effects of resveratrol and SIRT1 activation on dystrophic cardiomyopathy. Ann N. Y Acad Sci. 2015;1348:46–54. 26109180 10.1111/nyas.12812

[22] Sebori R, Kuno A, Hosoda R, Hayashi T, Horio Y. Resveratrol decreases oxidative stress by restoring mitophagy and improves the pathophysiology of dystrophin-deficient mdx mice. Oxid Med Cell Longev. 2018;2018:9179270. 30510631 10.1155/2018/9179270 PMC6231358

[23] Walle T, Hsieh F, DeLegge MH, Oatis JE Jr., Walle UK. High absorption but very low bioavailability of oral resveratrol in humans. Drug Metab Dispos. 2004;32:1377–82. 15333514 10.1124/dmd.104.000885

[24] Gambini J, Ingles M, Olaso G, Lopez-Grueso R, Bonet-Costa V, Gimeno-Mallench L, et al. Properties of resveratrol: in vitro and in vivo studies about metabolism, bioavailability, and biological effects in animal models and humans. Oxid Med Cell Longev. 2015;2015:837042. 26221416 10.1155/2015/837042 PMC4499410

[25] Ung D, Nagar S. Variable sulfation of dietary polyphenols by recombinant human sulfotransferase (SULT) 1A1 genetic variants and SULT1E1. Drug Metab Dispos. 2007;35:740–6. 17293380 10.1124/dmd.106.013987

[26] Mehboob H, Tahir IM, Iqbal T, Saleem S, Perveen S, Farooqi A. Effect of UDP-Glucuronosyltransferase (UGT) 1A Polymorphism (rs8330 and rs10929303) on Glucuronidation Status of Acetaminophen. Dose-response. 2017;15:1559325817723731. 28932176 10.1177/1559325817723731 PMC5598801

[27] Chang N, Li J, Lin S, Zhang J, Zeng W, Ma G, et al. Emerging roles of SIRT1 activator, SRT2104, in disease treatment. Sci Rep. 2024;14. 5521. 38448466 10.1038/s41598-024-55923-8 PMC10917792

[28] Hoffmann E, Wald J, Lavu S, Roberts J, Beaumont C, Haddad J, et al. Pharmacokinetics and tolerability of SRT2104, a first-in-class small molecule activator of SIRT1, after single and repeated oral administration in man. Br J Clin Pharmacol. 2013;75:186–96. 22616762 10.1111/j.1365-2125.2012.04340.x PMC3555058

[29] Liu H, Zhang Y, Zhang H, Xu S, Zhao H, Liu X. Abeta-INDUCED DAMAGE MEMory in hCMEC/D3 cells mediated by Sirtuin-1. Int J Mol Sci. 2020;21:8226. 10.3390/ijms21218226 PMC7662699 33153131

[30] Jiang M, Zheng J, Peng Q, Hou Z, Zhang J, Mori S, et al. Sirtuin 1 activator SRT2104 protects Huntington’s disease mice. Annals Clin Transl Neurol. 2014;1:1047–52. 10.1002/acn3.135 PMC4284130 25574479

[31] Yang J, Wang N, Zhu Y, Feng P. Roles of SIRT1 in high glucose-induced endothelial impairment: association with diabetic atherosclerosis. Arch Med Res. 2011;42:354–60. 21810449 10.1016/j.arcmed.2011.07.005

[32] Krueger JG, Suarez-Farinas M, Cueto I, Khacherian A, Matheson R, Parish LC, et al. A Randomized, Placebo-Controlled Study of SRT2104, a SIRT1 activator, in patients with moderate to severe Psoriasis. PLoS ONE. 2015;10:e0142081. 26556603 10.1371/journal.pone.0142081 PMC4640558

[33] Sands BE, Joshi S, Haddad J, Freudenberg JM, Oommen DE, Hoffmann E, et al. Assessing colonic exposure, safety, and clinical activity of SRT2104, a novel oral SIRT1 activator, in patients with mild to moderate ulcerative colitis. Inflamm Bowel Dis. 2016;22:607–14. 26595549 10.1097/MIB.0000000000000597 PMC4885523

[34] Abe-Higuchi N, Uchida S, Yamagata H, Higuchi F, Hobara T, Hara K, et al. Hippocampal Sirtuin 1 signaling mediates depression-like behavior. Biol Psychiatry. 2016;80:815–26. 27016384 10.1016/j.biopsych.2016.01.009

[35] Wesolowski LT, Simons JL, Semanchik PL, Othman MA, Kim JH, Lawler JM, et al. The Impact of SRT2104 on skeletal muscle mitochondrial function, redox biology, and loss of muscle mass in hindlimb unloaded rats. Int J Mol Sci. 2023;24:11135. 10.3390/ijms241311135 PMC10342025 37446313

[36] Mercken EM, Mitchell SJ, Martin-Montalvo A, Minor RK, Almeida M, Gomes AP, et al. SRT2104 extends survival of male mice on a standard diet and preserves bone and muscle mass. Aging cell. 2014;13:787–96. 24931715 10.1111/acel.12220 PMC4172519

[37] van der Meer AJ, Scicluna BP, Moerland PD, Lin J, Jacobson EW, Vlasuk GP, et al. The selective Sirtuin 1 Activator SRT2104 reduces endotoxin-induced cytokine release and coagulation activation in humans. Crit Care Med. 2015;43:e199–202. 25978169 10.1097/CCM.0000000000000949

[38] Miglietta D, De Palma C, Sciorati C, Vergani B, Pisa V, Villa A, et al. Naproxcinod shows significant advantages over naproxen in the mdx model of Duchenne Muscular Dystrophy. Orphanet J Rare Dis. 2015;10:101. 26296873 10.1186/s13023-015-0311-0 PMC4546261

[39] Catalani E, Bongiorni S, Taddei AR, Mezzetti M, Silvestri F, Coazzoli M, et al. Defects of full-length dystrophin trigger retinal neuron damage and synapse alterations by disrupting functional autophagy. Cell Mol Life Sci. 2021;78:1615–36. 32749504 10.1007/s00018-020-03598-5 PMC7904721

[40] Zecchini S, Giovarelli M, Perrotta C, Morisi F, Touvier T, Di Renzo I, et al. Autophagy controls neonatal myogenesis by regulating the GH-IGF1 system through a NFE2L2- and DDIT3-mediated mechanism. Autophagy. 2019;15:58–77. 30081710 10.1080/15548627.2018.1507439 PMC6287695

[41] Thorley M, Duguez S, Mazza EMC, Valsoni S, Bigot A, Mamchaoui K, et al. Skeletal muscle characteristics are preserved in hTERT/cdk4 human myogenic cell lines. Skelet Muscle. 2016;6:43. 27931240 10.1186/s13395-016-0115-5 PMC5146814

[42] Mocciaro E, Giambruno R, Micheloni S, Cernilogar FM, Andolfo A, Consonni C, et al. WDR5 is required for DUX4 expression and its pathological effects in FSHD muscular dystrophy. Nucleic Acids Res. 2023;51:5144–61. 37021550 10.1093/nar/gkad230 PMC10250208

[43] Neuman S, Kovalio M, Yaffe D, Nudel U. The Drosophila homologue of the dystrophin gene - introns containing promoters are the major contributors to the large size of the gene. FEBS Lett. 2005;579:5365–71. 16198353 10.1016/j.febslet.2005.08.073

[44] Cervia D, Zecchini S, Pincigher L, Roux-Biejat P, Zalambani C, Catalani E, et al. Oral administration of plumbagin is beneficial in in vivo models of Duchenne muscular dystrophy through control of redox signaling. Free Radic Biol Med. 2024;225:193–207. 39326684 10.1016/j.freeradbiomed.2024.09.037

[45] Shcherbata HR, Yatsenko AS, Patterson L, Sood VD, Nudel U, Yaffe D, et al. Dissecting muscle and neuronal disorders in a Drosophila model of muscular dystrophy. EMBO J. 2007;26:481–93. 17215867 10.1038/sj.emboj.7601503 PMC1783456

[46] Yoshimoto Y, Ikemoto-Uezumi M, Hitachi K, Fukada SI, Uezumi A. Methods for accurate assessment of myofiber maturity during skeletal muscle regeneration. Front Cell Dev Biol. 2020;8:267. 32391357 10.3389/fcell.2020.00267 PMC7188918

[47] Giovarelli M, Zecchini S, Martini E, Garre M, Barozzi S, Ripolone M, et al. Drp1 overexpression induces desmin disassembling and drives kinesin-1 activation promoting mitochondrial trafficking in skeletal muscle. Cell Death Differ. 2020;27:2383–401. 10.1038/s41418-020-0510-7 PMC7370230 32042098

[48] Reggio A, Rosina M, Krahmer N, Palma A, Petrilli LL, Maiolatesi G. et al. Metabolic reprogramming of fibro/adipogenic progenitors facilitates muscle regeneration. Life Sci. Alliance. 2020;3:e202000646 32019766 10.26508/lsa.202000660 PMC7003708

[49] Liang D, Chen C, Huang S, Liu S, Fu L, Niu Y. Alterations of Lysine Acetylation profile in murine skeletal muscles upon exercise. Front Aging Neurosci. 2022;14:859313. 35592697 10.3389/fnagi.2022.859313 PMC9110802

[50] Rybalka E, Timpani CA, Cooke MB, Williams AD, Hayes A. Defects in mitochondrial ATP synthesis in dystrophin-deficient mdx skeletal muscles may be caused by complex I insufficiency. PLoS ONE. 2014;9:e115763. 25541951 10.1371/journal.pone.0115763 PMC4277356

[51] Meyer P, Notarnicola C, Meli AC, Matecki S, Hugon G, Salvador J, et al. Skeletal Ryanodine receptors are involved in impaired myogenic differentiation in duchenne muscular dystrophy patients. Int J Mol Sci. 2021;22:12985. 10.3390/ijms222312985 PMC8657486 34884796

[52] Ryall JG, Dell’Orso S, Derfoul A, Juan A, Zare H, Feng X, et al. The NAD(+)-dependent SIRT1 deacetylase translates a metabolic switch into regulatory epigenetics in skeletal muscle stem cells. Cell Stem Cell. 2015;16:171–83. 25600643 10.1016/j.stem.2014.12.004 PMC4320668

[53] Careccia G, Mangiavini L, Cirillo F. Regulation of satellite cells functions during skeletal muscle regeneration: a critical step in physiological and pathological conditions. Int J Mol Sci. 2023;25:512. 10.3390/ijms25010512 PMC10778731 38203683

[54] Hernandez-Hernandez JM, Garcia-Gonzalez EG, Brun CE, Rudnicki MA. The myogenic regulatory factors, determinants of muscle development, cell identity and regeneration. Semin Cell Dev Biol. 2017;72:10–8. 29127045 10.1016/j.semcdb.2017.11.010 PMC5723221

[55] Fujiwara D, Iwahara N, Sebori R, Hosoda R, Shimohama S, Kuno A, et al. SIRT1 deficiency interferes with membrane resealing after cell membrane injury. PLoS ONE. 2019;14:e0218329. 31242212 10.1371/journal.pone.0218329 PMC6594621

[56] Chalkiadaki A, Igarashi M, Nasamu AS, Knezevic J, Guarente L. Muscle-specific SIRT1 gain-of-function increases slow-twitch fibers and ameliorates pathophysiology in a mouse model of duchenne muscular dystrophy. PLoS Genet. 2014;10:e1004490. 25032964 10.1371/journal.pgen.1004490 PMC4102452

[57] Domi E, Hoxha M, Prendi E, Zappacosta B. A systematic review on the role of SIRT1 in Duchenne muscular dystrophy. Cells. 2023;16:dmm049930. 10.3390/cells10061380 PMC8229470 34205021

[58] Ljubicic V, Burt M, Lunde JA, Jasmin BJ. Resveratrol induces expression of the slow, oxidative phenotype in mdx mouse muscle together with enhanced activity of the SIRT1-PGC-1alpha axis. Am J Physiol Cell Physiol. 2014;307:C66–82. 24760981 10.1152/ajpcell.00357.2013 PMC4080183

[59] Gordon BS, Delgado Diaz DC, Kostek MC. Resveratrol decreases inflammation and increases utrophin gene expression in the mdx mouse model of Duchenne muscular dystrophy. Clinical Nutr. 2013;32:104–11. 10.1016/j.clnu.2012.06.003 22795790

[60] Capogrosso RF, Cozzoli A, Mantuano P, Camerino GM, Massari AM, Sblendorio VT, et al. Assessment of resveratrol, apocynin and taurine on mechanical-metabolic uncoupling and oxidative stress in a mouse model of duchenne muscular dystrophy: a comparison with the gold standard, alpha-methyl prednisolone. Pharmacol Res. 2016;106:101–13. 26930420 10.1016/j.phrs.2016.02.016

[61] Gordon BS, Delgado-Diaz DC, Carson J, Fayad R, Wilson LB, Kostek MC. Resveratrol improves muscle function but not oxidative capacity in young mdx mice. Can J Physiol Pharm. 2014;92:243–51. 10.1139/cjpp-2013-0350 24593789

[62] Hou X, Rooklin D, Fang H, Zhang Y. Resveratrol serves as a protein-substrate interaction stabilizer in human SIRT1 activation. Scientific Rep. 2016;6. 38186. 10.1038/srep38186 PMC5128864 27901083

[63] Curry AM, White DS, Donu D, Cen Y. Human Sirtuin Regulators: the “Success” Stories. Front Physiol. 2021;12. 752117. 34744791 10.3389/fphys.2021.752117 PMC8568457

[64] Springer M, Moco S. Resveratrol and its human metabolites-effects on metabolic health and obesity. Nutrients. 2019;11:143. 10.3390/nu11010143 PMC6357128 30641865

[65] Calamini B, Ratia K, Malkowski MG, Cuendet M, Pezzuto JM, Santarsiero BD, et al. Pleiotropic mechanisms facilitated by resveratrol and its metabolites. Biochem J. 2010;429:273–82. 20450491 10.1042/BJ20091857 PMC3265359

[66] Libri V, Brown AP, Gambarota G, Haddad J, Shields GS, Dawes H, et al. A pilot randomized, placebo controlled, double blind phase I trial of the novel SIRT1 activator SRT2104 in elderly volunteers. PLoS ONE. 2012;7:e51395. 23284689 10.1371/journal.pone.0051395 PMC3527451

[67] Noh RM, Venkatasubramanian S, Daga S, Langrish J, Mills NL, Lang NN, et al. Cardiometabolic effects of a novel SIRT1 activator, SRT2104, in people with type 2 diabetes mellitus. Open Heart. 2017;4:e000647. 28912956 10.1136/openhrt-2017-000647 PMC5588958

[68] Baksi A, Kraydashenko O, Zalevkaya A, Stets R, Elliott P, Haddad J, et al. A phase II, randomized, placebo-controlled, double-blind, multi-dose study of SRT2104, a SIRT1 activator, in subjects with type 2 diabetes. British J Clin Pharmacol. 2014;78:69–77. 10.1111/bcp.12327 PMC4168381 24446723

[69] Yang H, Tang L, Qu Z, Lei SH, Li W, Wang YH. Hippocampal insulin resistance and the Sirtuin 1 signaling pathway in diabetes-induced cognitive dysfunction. Neural Regener Res. 2021;16:2465–74. 10.4103/1673-5374.313051 PMC8374594 33907035

[70] Gupta MP, Samant SA, Smith SH, Shroff SG. HDAC4 and PCAF bind to cardiac sarcomeres and play a role in regulating myofilament contractile activity. J Biol Chem. 2008;283:10135–46. 18250163 10.1074/jbc.M710277200 PMC2442284

[71] Russell B, Solis C. Mechanosignaling pathways alter muscle structure and function by post-translational modification of existing sarcomeric proteins to optimize energy usage. J Muscle Res Cell Motil. 2021;42:367–80. 33595762 10.1007/s10974-021-09596-9 PMC8338793

[72] Wang Q, Zhang Y, Yang C, Xiong H, Lin Y, Yao J, et al. Acetylation of metabolic enzymes coordinates carbon source utilization and metabolic flux. Science. 2010;327:1004–7. 20167787 10.1126/science.1179687 PMC4183141

[73] Zhang ZH, Zhang H, Wang YR, Liu XL, Huang H, Xu XH. SIRT 1 binding with PKM and NSE and modulate their acetylation and activities. Biochim et Biophys Acta Proteins Proteom. 2019;1867:794–801. 10.1016/j.bbapap.2019.06.003 31202897

[74] Walker MA, Chavez J, Villet O, Tang X, Keller A, Bruce JE, et al. Acetylation of muscle creatine kinase negatively impacts high-energy phosphotransfer in heart failure. JCI insight. 2021;6:e144301. 10.1172/jci.insight.144301 PMC7934860 33554956

[75] Boccanegra B, Mantuano P, Conte E, Cerchiara AG, Tulimiero L, Quarta R, et al. LKB1 signaling is altered in skeletal muscle of a Duchenne muscular dystrophy mouse model. Dis Models Mech. 2023;16. 10.1242/dmm.049930 PMC10354716 37427454

[76] Pambianco S, Giovarelli M, Perrotta C, Zecchini S, Cervia D, Di Renzo I, et al. Reversal of defective mitochondrial biogenesis in limb-girdle muscular dystrophy 2D by independent modulation of histone and PGC-1alpha acetylation. Cell Rep. 2016;17:3010–23. 27974213 10.1016/j.celrep.2016.11.044

[77] Feige JN, Lagouge M, Canto C, Strehle A, Houten SM, Milne JC, et al. Specific SIRT1 activation mimics low energy levels and protects against diet-induced metabolic disorders by enhancing fat oxidation. Cell Metab. 2008;8:347–58. 19046567 10.1016/j.cmet.2008.08.017

[78] Panza E, Vellecco V, Iannotti FA, Paris D, Manzo OL, Smimmo M, et al. Duchenne’s muscular dystrophy involves a defective transsulfuration pathway activity. Redox Biol. 2021;45:102040. 34174560 10.1016/j.redox.2021.102040 PMC8246642

[79] Kazirod K, Myszka M, Dulak J, Loboda A. Hydrogen sulfide as a therapeutic option for the treatment of Duchenne muscular dystrophy and other muscle-related diseases. Cell Mol Life Sci. 2022;79:608. 36441348 10.1007/s00018-022-04636-0 PMC9705465

[80] Saclier M, Ben Larbi S, My Ly H, Moulin E, Mounier R, Chazaud B, et al. Interplay between myofibers and pro-inflammatory macrophages controls muscle damage in mdx mice. J Cell Sci. 2021;134:jcs258429. 10.1242/jcs.258429 34471933

[81] Ellwood RA, Hewitt JE, Torregrossa R, Philp AM, Hardee JP, Hughes S, et al. Mitochondrial hydrogen sulfide supplementation improves health in the C. elegans Duchenne muscular dystrophy model. Proc Natl Acad Sci USA. 2021;118:e2018342118. 10.1073/pnas.2018342118 PMC7936346 33627403

[82] Timpani CA, Hayes A, Rybalka E. Revisiting the dystrophin-ATP connection: how half a century of research still implicates mitochondrial dysfunction in Duchenne Muscular Dystrophy aetiology. Med Hypotheses. 2015;85:1021–33. 26365249 10.1016/j.mehy.2015.08.015

[83] Mantuano P, Sanarica F, Conte E, Morgese MG, Capogrosso RF, Cozzoli A, et al. Effect of a long-term treatment with metformin in dystrophic mdx mice: a reconsideration of its potential clinical interest in Duchenne muscular dystrophy. Biochem Pharm. 2018;154:89–103. 29684379 10.1016/j.bcp.2018.04.022

