# Supplementary material for: The SIRT1 activator SRT2104 exerts exercise mimetic effects and promotes Duchenne muscular dystrophy recovery
Source: Cell Death Dis. 2025 Apr 7;16(1):259. doi: 10.1038/s41419-025-07595-z (PMC11977210; doi:10.1038/s41419-025-07595-z)
Supplement: Supplementary file 1 — Supplementary material [file 41419_2025_7595_MOESM1_ESM.pdf]

Supplementary Materials

Materials and Methods

Nucleic acid extractions, quantitative RT-qPCR.

Total RNA was isolated from Tibialis Anterior (TA), DP, and human myoblasts using PureZOL reagent (Bio-Rad, Hercules, CA, USA) following the manufacturer's instructions. Total RNA (500 ng) was retrotranscribed using the iScript Reverse Transcription Supermix (Bio-Rad, Hercules, CA, USA). RT-qPCR was performed using the SsoAdvanced Universal SYBR Green Supermix (Bio-Rad, Hercules, CA, USA) and the CFX96 Touch Real-Time PCR Detection System (Bio-Rad). All reactions were run as duplicates and the fold changes were determined relative to the 36B4 housekeeping transcripts using the $2^{-\Delta\Delta CT}$ formula.

Mitochondrial DNA (mtDNA) from mice samples was quantified as already described (10). 20 ng of DNA per sample was used for each amplification reaction. The mtDNA level per nuclear genome was measured by qPCR using specific primers for Cytochrome B and RNase P. mtDNA quantification of the relative copy number per nuclear DNA (nuDNA) was analyzed using the $2^{-\Delta\Delta CT}$ formula.

In Supplementary Table 1 the list of primers used is reported (Eurofin genomics, Ebersberg, Germany; Bio-Rad, Hercules, CA, USA). For human samples, custom BioRad primes [qHsaCED0044251 (MYOD); qHsaCED0022985 (MYOG)] have been used.

Protein isolation and Western Blotting

Tissue samples from TA and GS were homogenized through Ultra-Turrax (Ika-lab, Staufen, Germany) in lysis buffer containing 20 mM Tris-HCl (pH 7.4), 10 mM EGTA, 150 mM NaCl, 1% Triton X-100, 10% glycerol, SDS 2% supplemented with a cocktail of protease and phosphatase inhibitors (cOmplete and PhosSTOP; Roche Applied Science Mannheim, Germany). Human myoblast lysates were prepared with RIPA buffer supplemented with a cocktail of protease and phosphatase inhibitors. After centrifugation at 10,000 × g for 10 min, proteins were quantified by DC Protein Assay (Bio-Rad, Hercules, CA, USA). 30 to 50 µg of total protein were loaded on 4–20% polyacrylamide precast gels (Criterion TGX Stain-free precast gels; Bio-Rad, Hercules, CA, USA) and were transferred onto a nitrocellulose membrane using a Trans-Blot Turbo SystemTM and Transfer packTM (Bio-Rad, Hercules, CA, USA). The membranes were probed using the primary antibodies listed in Supplementary Table 2. The bands were visualized using horseradish-peroxidase-conjugated secondary antibodies (Bio-Rad) and the Clarity Western ECL Substrate with ChemiDocMP Imaging System (Bio-Rad, Hercules, CA, USA). Blots were routinely treated with glycine (0.2M pH 2.5) stripping buffer and reprobed with the appropriate antibodies. Results were analyzed

using the Image Lab software (Bio-Rad, Hercules, CA, USA). Full-length uncropped original western blots are provided as a single supplementary file.

High-resolution respirometry (HRR)

Upon mice sacrifice, muscles were cryopreserved in cryotubes containing 1 mL of ice-cold modified University of Wisconsin solution (20mM histidine, 20mM succinate, 3mM glutathione, 1 μM leupeptin, 2mM glutamate, 2mM malate, 2mM ATP, 0.5mM EGTA, 3mM MgCl2•6H2O, 60mM MOPS, 20mM taurine, 10mM KH2PO4, 20mM HEPES, 110mM sucrose, 1 g/L BSA and 10% (v/v) DMSO). DMSO was added immediately before cryopreservation The biopsies were cryopreserved following a sequential freezing method as previously described (1). Since cryopreservation implies freezing and thawing, the samples did not require permeabilization with chemical agents. Oxygen consumption rates were measured by high-resolution respirometry on DP bundles from vehicles and SRT2104-treated *mdx* mice. The respiration rates of fibers (1–3 mg wet weight) were measured in the O2K oxygraph chambers (Instruments Oroboros, Innsbruck, Austria) at 37 °C in a respiration medium MiR06 (0.5 mM EGTA, 3 mM MgCl2, 60 mM K-lactobionate, 20 mM taurine, 10 mM KH2PO4, 20 mM Hepes, 110 mM sucrose and 1 g/l bovine serum albumin fatty acid-free, 280 U/ml catalase (pH 7.1)). To detect the electron flow through respiratory chain complexes, substrates, uncouplers, and inhibitors were sequentially added as previously described (10). Glutamate (10 mM) and malate (2 mM) are injected to obtain state LEAK state. Then we added ADP (2.5 mM) revealing complex I (CI) oxidative phosphorylation capacity (State 3). The addition of cytochrome C (10 μM) was performed to test the integrity of the outer mitochondrial membrane and provided the quality control of permeabilized fibers. Subsequent titration of succinate (10 mM) led to the evaluation of state 3 by convergent electron flow from both complexes I and II thus obtaining the maximal OXPHOS capacity (CI+II). The maximal capacity of the Electron Transfer System (ETS) was evaluated by a 0.5 μM steps titration of the uncoupler protonophore carbonyl cyanide p-trifluoro- methoxyphenyl hydrazone (FCCP). Uncoupled complex-II-linked respiration was achieved by the addition of rotenone (0.5 μM) (CII) and finally, the respiratory system was inhibited with the complex II inhibitor Malonate (5 mM) and the complex III inhibitor antimycin A (2.5 μM) to obtain the non-mitochondrial residual oxygen consumption flux (ROX). Complex IV (CIV) activity was stimulated by using N,N,N0,N0-Tetramethyl-p-phenyl-enediamine dihydrochloride (TMPD) (0.5 μM) and ascorbate (2 mM), recorded for 5 min and hence stopped with the addition of CIV inhibitor sodium azide (100 mM) to calculate the TMPD autoxidation oxygen consumption. Oxygen fluxes were corrected by subtracting residual oxygen consumption flux (ROX) from each steady state. The DatLab7 software (Instruments Oroboros, Innsbruck, Austria) was used for data acquisition and analysis.

Measurement of ATP formation

A luminescence assay (CellTiter-Glo Luminescent Cell Viability Assay, Promega, Madison, Wisconsin, USA) was used to determine ATP content in gastrocnemius of *mdx* mice treated or not with SRT2104. Specifically, frozen tissues were homogenized in 0.3 ml of cold lysis buffer (0.25 M sucrose, 10 mM HEPES-NaOH pH 7.4), with ultra-turrax (10 s at max speed), and the homogenates were cleared by centrifugation at 1,000xg, at 4°C for 10 min. 250 µl of supernatant was quickly added to an equal volume of ice-cold 10% trichloroacetic acid (TCA), shaken for 20 s, and then centrifuged 10 min at 10.000xg at 4°C. After TCA extraction, TCA was neutralized by adding 200 µl of Tris-acetate buffer (1 M pH 8) to 400 µl of supernatant. Following a 10-fold dilution with deionized water, the extract was used for luciferin-luciferase assay. The reaction mix, containing luciferase and substrate, was added and the light emission was measured using a GloMax luminometer (Promega, Madison, Wisconsin, USA) and quantified according to an ATP standard curve.

Respiratory chain complexes activity

30–50 mg of GC muscles were minced in small fragments and diluted 1:10 in ice-cold sucrose muscle homogenization buffer in a 1 ml glass-glass tissue grinder. Muscle was then homogenized using a clean glass-glass conical tissue grinder kept on ice with 15 slow and controlled up-down strokes. Muscle homogenate was then centrifuged at 800g for 10 min at 4 °C and the supernatant was used for the Respiratory Chain (RC) analysis. Spectrophotometric assays of individual respiratory complex activities were carried out on tissue homogenates by using a single-wavelength spectrophotometer. Specific activities of each complex were normalized to that of citrate synthase (CS), an index of mitochondrial mass. Protein concentrations were quantified by DC Protein Assay (Bio-Rad, Hercules, CA, USA).

Molecular dynamics and docking simulation

The starting structures of mouse (Mus Musculus, NCBI Reference Sequence: NP_062786.1) SIRT1 used in the present work have been obtained adapting the mouse sequence (i.e., an Ile/Leu substitution of the amino acid in position 360) on the experimentally resolved structures of human Sirt1 from the RCSB Protein Data Bank, i.e., PDB ID: 4zzh (2) for the open (inactive) form; PDB ID: 5btr for the closed (active) form. The deposited structures did not include NAD (+) molecules, hence their positions have been reconstructed based on the position in the catalytic domain (CD) of human Sirt1 (PDB ID: 4kxq) (3). For the present work six distinct systems have been simulated using Molecular Dynamics (MD) simulations (see further for details): a) the apo forms of the open and closed conformations of Sirt1 (oS1, cS1); b) the holo forms of the open and closed conformations of Sirt1 complexed with SRT2104 (oS1-SRT, cS1-SRT);

c) the apo and holo forms of the final configurations after MD simulations of the open conformation of Sirt1 (cS1*, cS1-SRT*). The scheme of the simulated systems and their possible interconversion pattern are reported in Fig. S4B. The structures of the SIRT1-SRT2104 complexes have been obtained through data-driven docking using the HADDOCK software (4), i.e., assuming the binding sites of SRT2104 are the same one of the other STACs and that the orientation of the thiazole-amide moiety is retained during the binding and considering the lowest energy structures obtained.

The parametrization for Molecular Dynamics simulations of SRT2104 and of NADP+ has been realized by Self Consistent Field (SCF) restricted Hartree–Fock (RHF) calculations performed in NWChem as implemented in the BiKi suite (5). The molecular wavefunctions and the corresponding electrostatic potentials have been computed with a 6-31G* basis set used for all the atoms included in the structure, with a total of 720 wavefunctions for SRT2104 and 520 wavefunctions for NADP(+). The quantum mechanical (QM) electrostatic potential of SRT2104 and NADP(+) has been computed using respectively 11048 and 8318 grid points, spaced by 0.5 A, a probe radius of 0.7 A and an atomic radius factor of 1. The RESP (Restrained ElectroStatic Potential) algorithm has been applied to the quantum mechanically (QM) calculated molecular electrostatic potential (ESP) at molecular surfaces using an atom-centered point charge model. The classical mechanical parameters for the molecules have been generated using the generalized Amber force field (GAFF) and adapted to the format of a standard residue topology in the Amber 99S-ILDN force field. The translation for the GROMACS package for molecular dynamics simulations has been performed using ACEPYPE.

All the systems considered for the present work have been parameterized in GROMACS 2020.7 using the AMBER99-SB ILDN force field, with the inclusion of an upgrade for the metal-coordinating residues (6). A parallelepipedal solvent box has been created around the protein, solvated with TIP3P water molecules (~ 22000). The net charge has been neutralized (with Na+ ions) and the electrostatics treated with the Particle Mesh Ewald (PME) scheme. After minimization with the steepest descent method (convergence:100 kJ mol–1 nm–1), the system was equilibrated (with isotropic positional restraints on protein heavy atoms, k = 1000 kJ mol–1 nm–2) for 2 ns in the NPT ensemble with p = 1 atm and T = 300 K, then for 2 ns in the NVT ensemble at T = 300 K. Eventually, we performed 100 ns of simulation for each system in the NVT ensemble employing a timestep of 2 fs and constraining all covalent bond lengths with the LINCS algorithm. Every simulation has been replicated three times to increase the statistics of sampling.

The analyses were performed using the GROMACS internal programs and routines with the support of in-house code and visually inspected using Visual Molecular Dynamics (VMD). The molecular graphics have been realized using Gnuplot (http://www.gnuplot.info/).

Determination of muscles acetylome landscape

TA muscles from *mdx* mice (n=5 for each condition) were smashed using ultra turrax homogenizers in lysis buffer containing 20 mM TRIS, 10 mM EGTA, 150mM sodium chloride, 1% Triton X-100, 10% glycerol, SDS 2% with the addition of protease inhibitors (Halt™ Protease Inhibitor Cocktail (100X), Thermo Scientific, Waltham, MA USA) and phosphatase inhibitors (PhosStop, Merck, Darmstadt, Germany). The pellets were discarded, and the supernatants were subjected to DC Protein Assay (Bio-Rad, Hercules, CA, USA) to quantify the total protein content, using BSA as standard (Thermo Scientific, Waltham, MA USA). For label-free quantitative proteomics, 40 µg of total proteins from each sample were digested using the FASP (Filter Aided Sample Preparation) protocol (7). Specifically, peptide mixtures were desalted on homemade Stage Tips C18 and stored at -20°C. For the acetylome analysis, 1 mg of total proteins from each sample was digested using the FASP (Filter Aided Sample Preparation) protocol. Briefly, proteins were diluted to 200 µl with 0.1 M Tris/HCl pH 7.4. Cysteines were reduced with 50 µl of 0.1 M DTE (1,4-dithioerythritol) in Tris/HCl pH 7.4 and incubated at 95 °C for 1 min; then samples were centrifuged on Amicon Ultra-3K at 14,000 x g for 10 min with a solution of urea 8 M. Cysteines were alkylated with 100 µl 0.05 M IAA (iodoacetamide) in urea 8 M added on the filter and laid at room temperature for 5 min; samples were centrifuged on Amicon Ultra-3K at 14,000 x g for 10 min with a solution of urea 8 M and then incubated overnight at 37 °C in the presence of sequencing grade trypsin (Merck, Darmstadt, Germany) 1:50 (w/w), upon dilution of urea up to 2 M with 50 mM ammonium bicarbonate buffer.

Then the peptides were purified and desalted with OASIS PRIME HLB C18 cartridges (Waters, Milford MA). The samples were eluted in 1 ml (0.5 ml + 0.5 ml) of ACN (acetonitrile) and MeOH (methanol)/water 90/10 and dried under nitrogen. The dried peptide pellet was resuspended in 1.5 ml of Immunoaffinity Purification Bind buffer (IAP) from the PTMScan HS Acetyl-Lysine Motif kit from Cell Signaling Technology (Danvers, Massachusetts, USA Cat.#46784, CST Inc.). Resuspended peptides were directly loaded on the immunoaffinity magnetic beads and incubated at 4°C for 2 hours. Then, the supernatant, containing the unmodified peptides, was removed, while the beads-peptides complexes were washed four times with HS IAP Wash buffer and twice with water. Finally, the modified peptides were eluted using 100 µl of TFA 0.15%. The eluted peptides were subsequently desalted using home-made Stage Tips C18 and injected in a capillary chromatographic system (EASY-nLC™ 1000 Integrated Ultra High-Pressure Nano-HPLC System, Proxeon Biosystem, Odense, Denmark) for peptide separations on a 75 µm i.d. × 15 cm reverse phase silica capillary column, packed with 1.9 µm ReproSil-Pur 120 Å C18-AQ (Dr. Maisch GmbH, Germany).

A 100 min-gradient of eluents A (pure water with 0.1% v/v formic acid) and B (acetonitrile with 0.1% v/v formic acid) was used to achieve separation (from 5% to 40% of B in 88 min, 300 nL/min flow rate). MS analyses were performed using a Q-Exactive mass spectrometer (Thermo Scientific, Waltham, MA USA)

equipped with a nano-electrospray ion source (Proxeon Biosystems, Odense, Denmark). Each sample was analyzed in technical triplicates for label-free quantitative proteomics and duplicates for the acetylome analysis. Full scan spectra were acquired with the lock-mass option, resolution set to 70,000, and mass range from m/z 300 to 2000. The ten most intense ions (charge exclusion: unassigned, 1, 6-8, >8) were selected to be fragmented (ddMS2). MS/MS spectra were acquired with resolution set to 17,500, NCE set to 25 with an isolation window of 2 m/z. All MS/MS data were analyzed using Mascot (version 2.6, Matrix Science) search engine to search the mouse_proteome 20220525 (63,641 sequences; 28,558,480 residues). Searches were performed with the following settings: trypsin as proteolytic enzyme; 5 missed cleavages allowed; carbamidomethylation on cysteine as fixed modification; protein N-terminus-acetylation, methionine oxidation, acetylation on lysine residues as variable modifications; mass tolerance was set to 5 ppm and 0.02 Da for precursor and fragment ions, respectively.

All data were then examined by MaxQuant software (v. 1.6.1.0) (8) for label-free protein quantification based on the precursor intensity, using the above search parameters, except for ± 20 ppm for fragment ions mass tolerance. FDR (False Discovery Rate) of 1% was selected for both peptide and protein identification, with a minimum of two peptides per protein with at least one unique. The complete dataset of identified and quantified proteins was subjected to: log transformation; filtering for sites with at least 50% quantification rate in one or more conditions; imputation of missing values, both site- and condition-specific; centering of data across their median; detection of alterations by linear regression model to define significantly differently expressed proteins or acetylated sites, with a p-value < 0.05, as an adaptation from PhosR script (9). The statistical analysis and the subsequent hierarchical clustering were performed using an in-house developed R script. GO analyses were performed using David software, freely available (10, 11).

The 28 unique differentially abundant acetylation sites identified by the analysis are reported in Supplementary Table 3.

The mass spectrometry proteomics data have been deposited to the ProteomeXchange Consortium via the PRIDE (12) partner repository with the dataset identifier PXD054747 and 10.6019/PXD054747.

Determination of muscle proteome

GC muscles were lysed in a buffer containing urea 9M/20mM HEPES pH 8, sodium orthovanadate 100mM, and β-glycerophosphate 1M. Proteins were extracted, resuspended in 25 mmol/L NH_4HCO_3 containing 0.1% RapiGest (Waters Corporation, Milford, MA, USA), sonicated, and centrifuged at 13,000 rpm for 3 min. After 15 min of sample incubation at 80 °C, proteins were reduced with 5 mmol/L DTT at 60 °C for 15 min, and carbamidomethylated with 10 mmol/L iodoacetamide for 30 min at room temperature in the darkness. Digestion was achieved with sequencing grade trypsin (Promega, Milan, Italy) at 37 °C overnight,

and then, 2% TFA was added to inactivate trypsin and hydrolyze RapiGest. Tryptic peptides were recovered and used for label-free mass spectrometry analysis (LC-MSE), which was performed on a hybrid quadrupole time-of-flight mass spectrometer (SYNAPT-XS, Waters Corporation, Milford, MA, USA) coupled with an M-class UPLC system and equipped with a nanosource (Waters Corporation). Samples were injected into a Symmetry C18 nanoACQUITY trap column, 100 Å, 5 µm, 180 µm × 2 cm (Waters Corporation, Milford, MA, USA), and subsequently analyzed using the analytical column HSS T3 C18, 100 Å, 1.7 µm, 75 µm × 150 mm (Waters Corporation, Milford, MA, USA), at a flow rate of 300 nL/min by increasing the organic solvent B concentration from 3 to 40% over 90 min, using 0.1% v/v formic acid in water as reversed phase solvent A, and 0.1% v/v formic acid in acetonitrile as reversed phase solvent B. All the samples were analyzed in duplicate by LC-MSE as previously described (13) in ion mobility-enhanced data-independent acquisition (IMS-DIA). Briefly, data were collected at a constant collision energy of 6 eV in the low-energy MS mode, while in the high-energy mode, the fragmentation was performed by applying drift time-specific collision energies (14). Progenesis QIP v 4.1 (Nonlinear Dynamics) was applied for statistical analysis using a Uniprot mouse protein sequence database (v2017). The mass spectrometry proteomics data have been deposited to the ProteomeXchange Consortium via the PRIDE (12) partner repository with the dataset identifier PXD055878 and 10.6019/PXD055878

Proteomic data was analyzed for differential expression and enrichment analysis. Principal Component Analysis (PCA) was computed to check the quality of data and replicates; no batch correction techniques were necessary. Then, the replicates of each sample were averaged together. The Fold Change (FC) was computed using the vehicles as references. To identify the proteins that deviate from the distribution of our samples, hence differentially expressed, a histogram was created to represent the combined distribution of the FC and the False Discovery Rate (FDR) as follows: $-\log10(FDR) * \log2(FC)$. The extremities of the histogram distribution ($|x| >= 0.05$) were selected and classified as the differential expressed proteins. On those, we performed enrichment analysis using EnrichR (version 3.2, R version 4.3.1) with different Gene Ontology (GO) terms, including Molecular Function (2023), Cellular Component (2023), Biological Process (2023) and KEGG (2019, Mouse).

References

1. Giovarelli M, Serati A, Zecchini S, Guelfi F, Clementi E, Mando C. Cryopreserved placental biopsies maintain mitochondrial activity for high-resolution respirometry. Mol Med. 2023;29(1):45.
2. Dai H, Case AW, Riera TV, Considine T, Lee JE, Hamuro Y, et al. Crystallographic structure of a small molecule SIRT1 activator-enzyme complex. Nat Commun. 2015;6:7645.
3. Davenport AM, Huber FM, Hoelz A. Structural and functional analysis of human SIRT1. J Mol Biol. 2014;426(3):526-41.
4. Dominguez C, Boelens R, Bonvin AM. HADDOCK: a protein-protein docking approach based on biochemical or biophysical information. J Am Chem Soc. 2003;125(7):1731-7.
5. Decherchi S, Bottegoni G, Spitaleri A, Rocchia W, Cavalli A. BiKi Life Sciences: A New Suite for Molecular Dynamics and Related Methods in Drug Discovery. Journal of chemical information and modeling. 2018;58(2):219-24.
6. Macchiagodena M, Pagliai M, Andreini C, Rosato A, Procacci P. Upgraded AMBER Force Field for Zinc-Binding Residues and Ligands for Predicting Structural Properties and Binding Affinities in Zinc-Proteins. ACS omega. 2020;5(25):15301-10.
7. Wisniewski JR, Zougman A, Nagaraj N, Mann M. Universal sample preparation method for proteome analysis. Nat Methods. 2009;6(5):359-62.
8. Cox J, Neuhauser N, Michalski A, Scheltema RA, Olsen JV, Mann M. Andromeda: a peptide search engine integrated into the MaxQuant environment. J Proteome Res. 2011;10(4):1794-805.
9. Kim HJ, Kim T, Hoffman NJ, Xiao D, James DE, Humphrey SJ, et al. PhosR enables processing and functional analysis of phosphoproteomic data. Cell Rep. 2021;34(8):108771.
10. Huang da W, Sherman BT, Lempicki RA. Systematic and integrative analysis of large gene lists using DAVID bioinformatics resources. Nat Protoc. 2009;4(1):44-57.
11. Sherman BT, Hao M, Qiu J, Jiao X, Baseler MW, Lane HC, et al. DAVID: a web server for functional enrichment analysis and functional annotation of gene lists (2021 update). Nucleic Acids Res. 2022;50(W1):W216-W21.
12. Perez-Riverol Y, Bai J, Bandla C, Garcia-Seisdedos D, Hewapathirana S, Kamatchinathan S, et al. The PRIDE database resources in 2022: a hub for mass spectrometry-based proteomics evidences. Nucleic Acids Res. 2022;50(D1):D543-D52.
13. Brioschi M, Eligini S, Crisci M, Fiorelli S, Tremoli E, Colli S, et al. A mass spectrometry-based workflow for the proteomic analysis of in vitro cultured cell subsets isolated by means of laser capture microdissection. Anal Bioanal Chem. 2014;406(12):2817-25.
14. Distler U, Kuharev J, Navarro P, Tenzer S. Label-free quantification in ion mobility-enhanced data-independent acquisition proteomics. Nat Protoc. 2016;11(4):795-812.

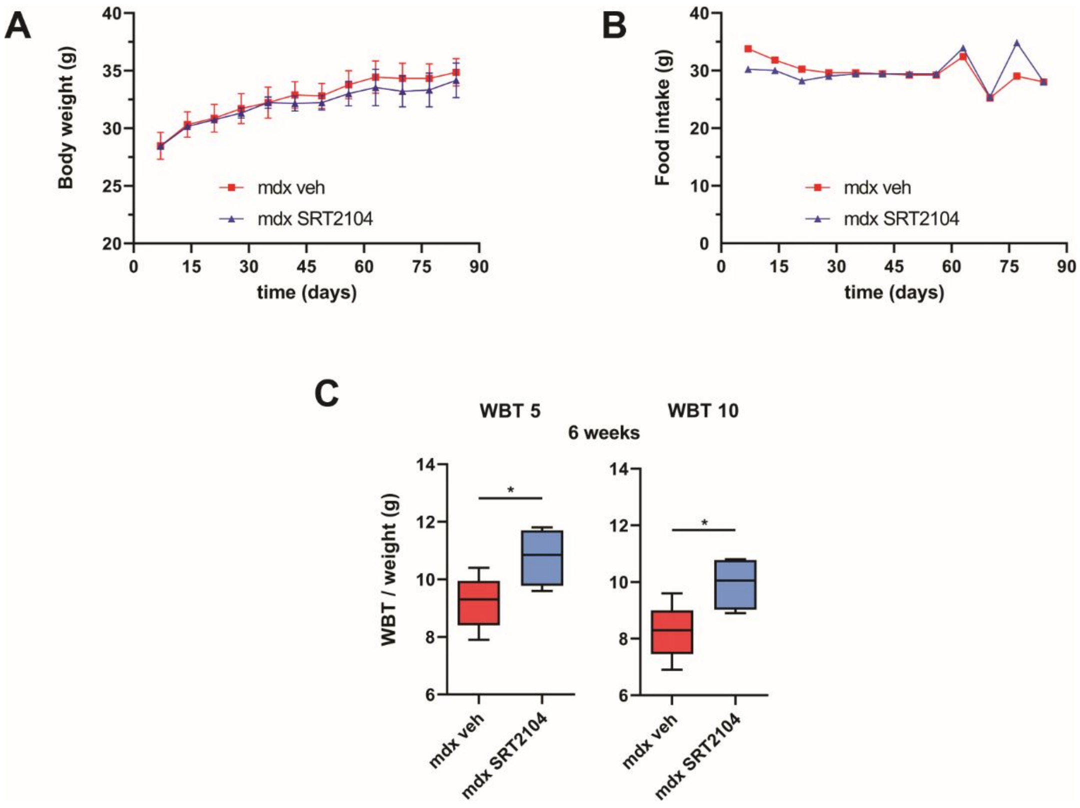

Figure S1. SRT2104 safety and mid-term benefits in *mdx* mice.

(**A-B**) Mean values of food intake (A) and body weight curve (B) of *mdx* mice treated with either vehicle or SRT2104 for 3 months (from 2 to 5 months of age). (**C**) WBT 5 and 10 measurements of *mdx* vehicle and SRT2104 evaluated at 3.5 months of age (at the midpoint of SRT2104 treatment) (*mdx* veh n=5; *mdx* SRT2104 n=4). * versus vehicle-treated *mdx* mice (* $P < 0.05$). Values are expressed as mean ± SEM.

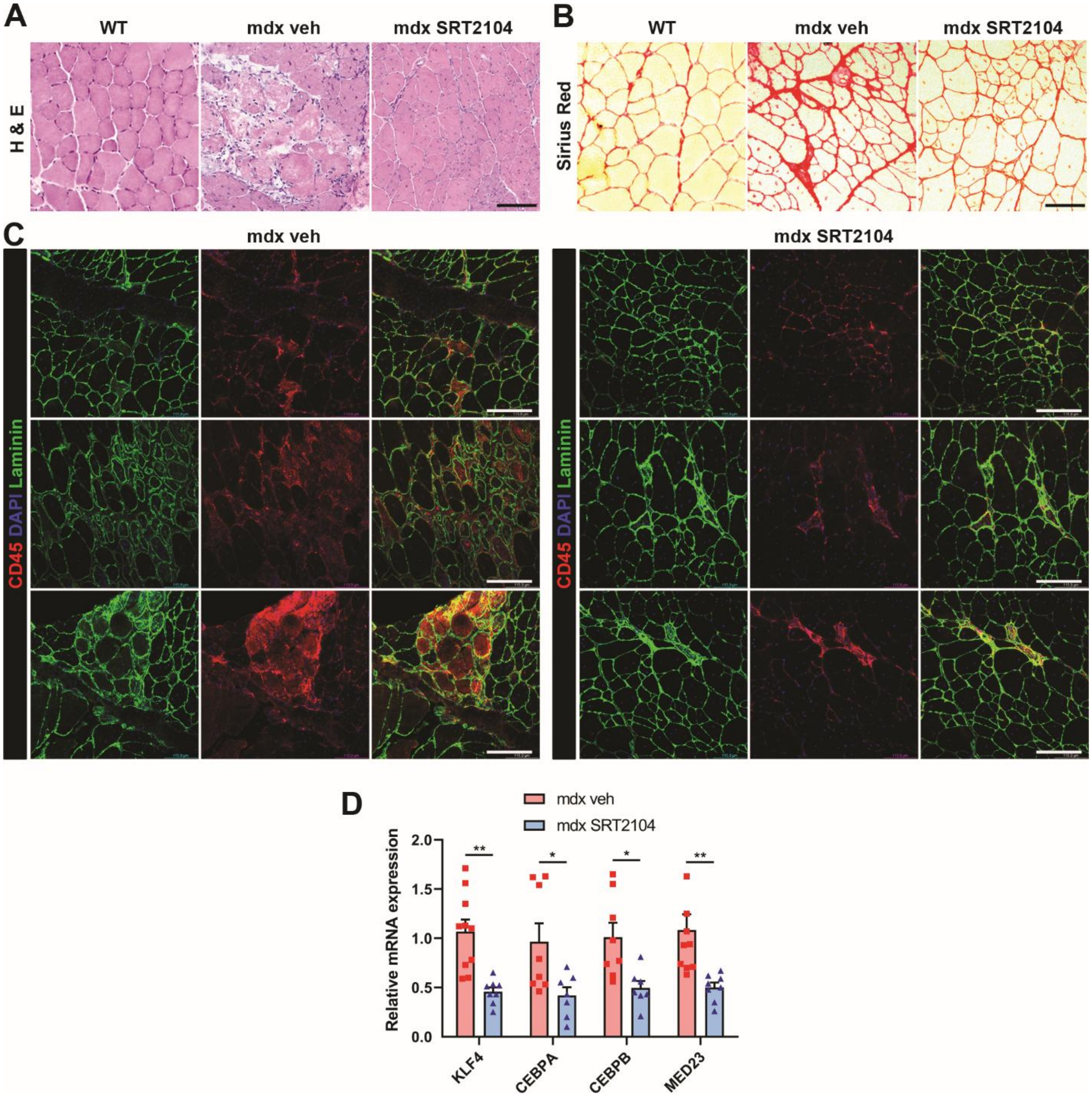

Figure S2. SRT2104 reduces adipogenic genes and improves TA muscle histology.

(**A-C**) Histological analyses of TA muscle of WT, and *mdx* mice treated or not with SRT2104: H&E (A), Sirius red (B), and CD45 staining (C) (scale bars=100 μm). (**D**) RT-qPCR analysis of adipogenic marker KLF4, CEBPA, CEBPB, and MED23 in DP of vehicle- and SRT2104-treated *mdx* mice (*mdx* veh n≥8; *mdx* SRT2104 n≥7).

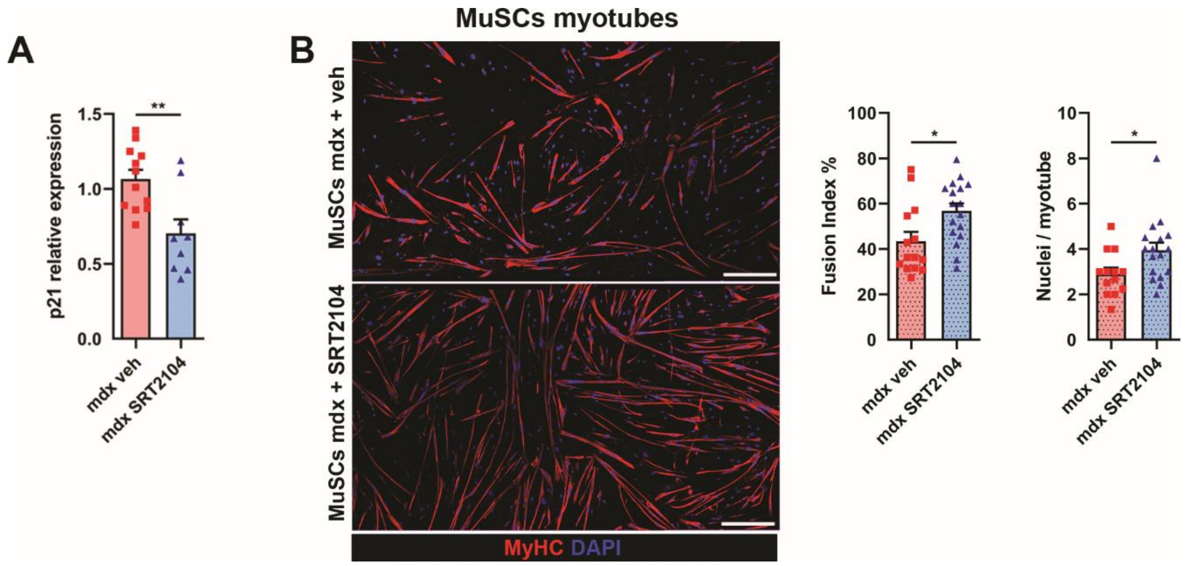

Figure S3. SRT2104 reduces p21 expression and increases myogenic potential of dystrophic MuSC-derived myotubes.

(**A**) RT-qPCR analysis of p21 gene in DP of *mdx* vehicle- and SRT2104-treated mice (*mdx* veh n=12; *mdx* SRT2104 n=9). * versus vehicle-treated *mdx* mice (** P < 0.01). (**B**) Representative myosin heavy chain immunostaining (red) and DAPI nuclear counterstaining (blue) of *mdx* satellite cells (MuSCs)-derived myotubes treated with either 3 μM SRT2104 or DMSO (vehicle) (scale bar=200 μm). Graphs showing the fusion index (calculated as the percentage of the number of nuclei within myotubes over the total number of nuclei) and the mean number of myonuclei per myotube are provided (the experiments were repeated 3 times; we analyzed at least 13 images for *mdx* veh and 17 images for *mdx* SRT2104). * versus vehicle-treated *mdx* MuSCs (* P < 0.05). Values are expressed as mean ± SEM.

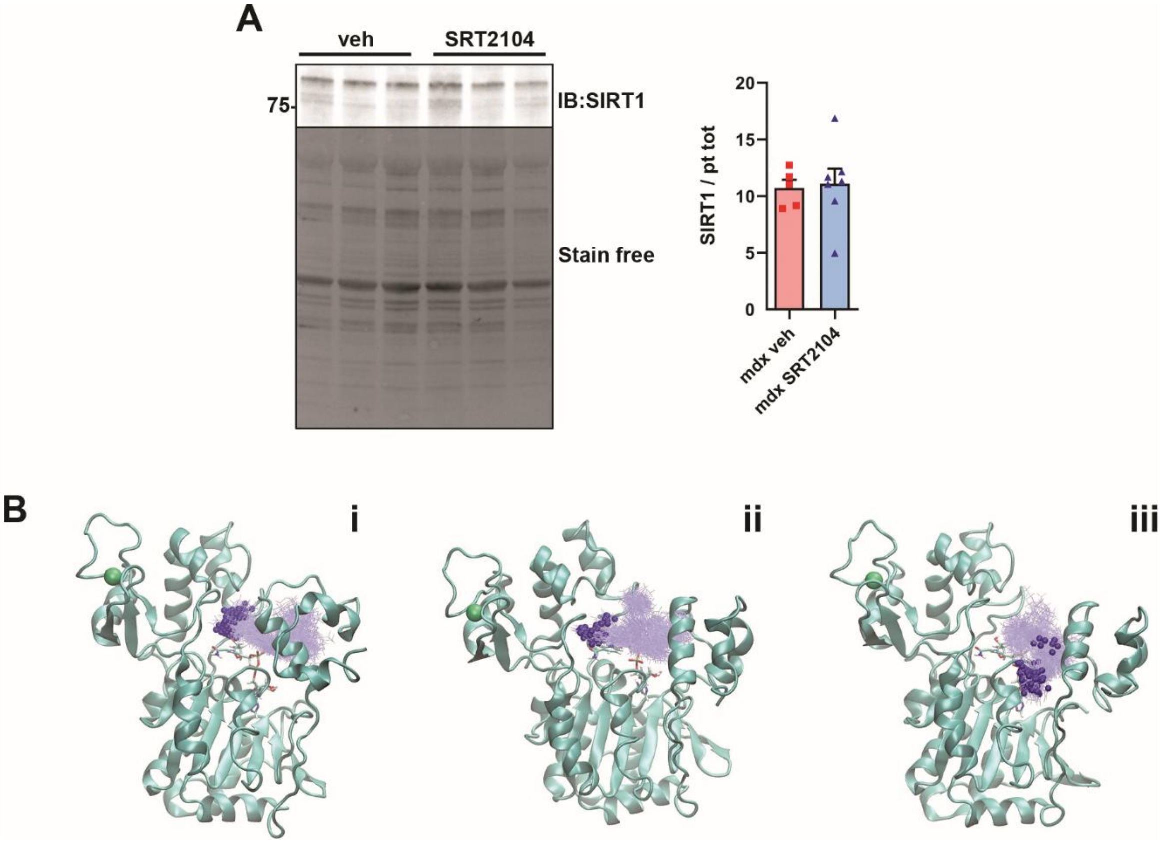

Figure S4. SIRT1 protein levels after SRT2104 treatment and MD simulation of SRT2104-SIRT1 binding mechanism.

(**A**) Representative immunoblot for SIRT1 in DP muscle protein extracts of *mdx* mice treated or not with SRT2104 normalized on stain-free total protein content. Densitometric quantification is provided (right panel) (*mdx* veh n=5; *mdx* SRT2104 n=7) Molecular weight (kDa) is indicated on the left. (**B**) Bundles of structures of cS1-SRT* complexes as identified by cluster analysis of the MD trajectories. The ones reported in i and ii cover the 80% of the population (respectively 40% and 40%), whereas the ones reported in iii cover the 20% of the population. The Zn atom is represented as a green sphere, the NAD molecule is represented in sticks with atomic CPK coding, a bundle of 20 SRT2104 structures is represented in light violet lines and the corresponding pyridinic nitrogen atom is represented as a dark violet sphere.

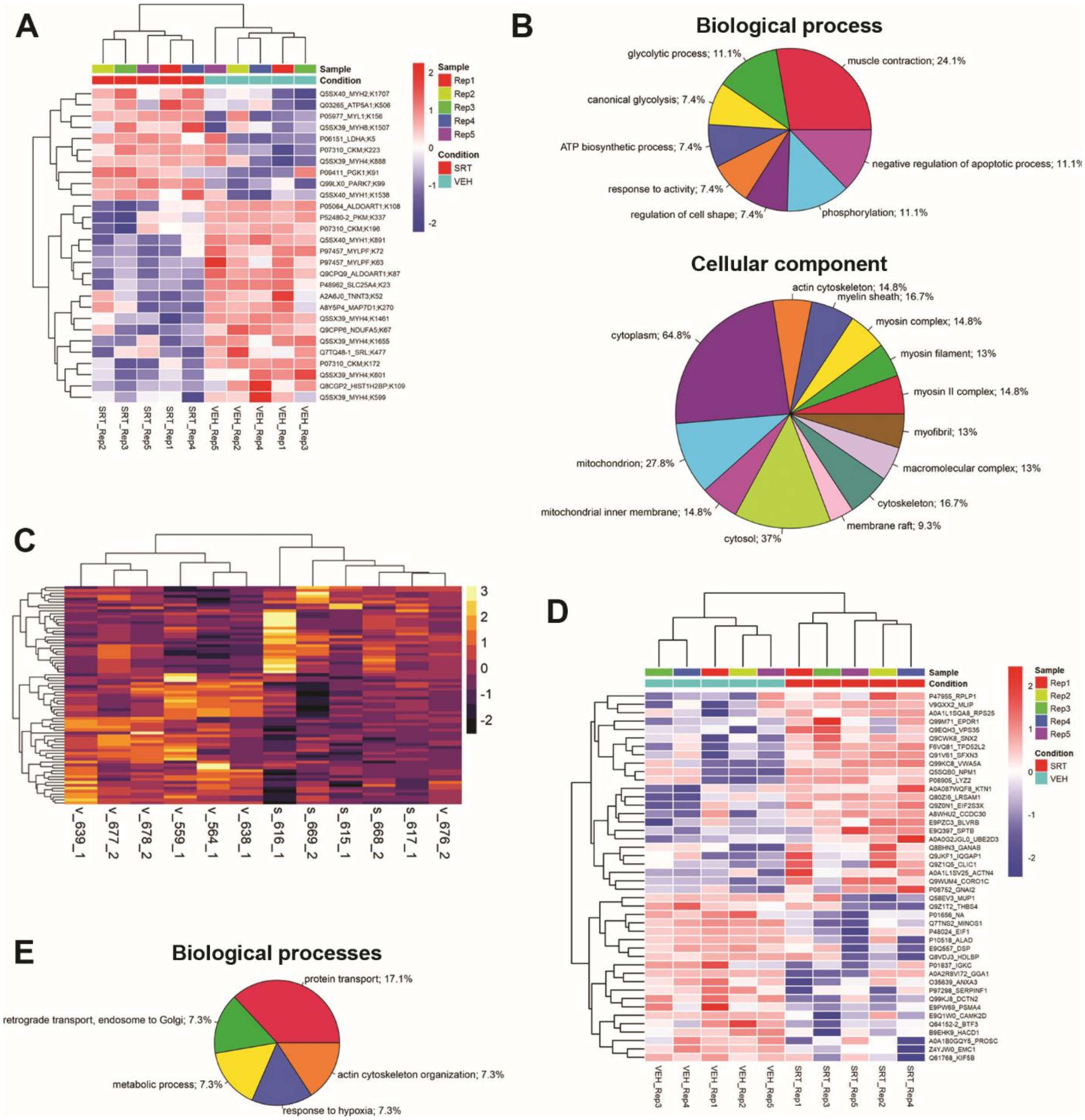

Figure S5. Acetylated protein and proteomic landscapes induced by SRT2104 in muscle of *mdx* mice.

(**A**) Site-specific heatmap of the 28 differentially acetylated proteins in TA muscle between vehicle- and SRT2104-treated *mdx* mice. (**B**) Pie charts of GO terms enrichment analyses in TA muscle showing differentially acetylated protein enrichments relative to Biological processes (upper panel) and Cellular components (lower panel). (**C**) Cluster analysis heatmap of the 74 differentially abundant proteins after proteomic analysis in dystrophic GC muscle treated or not with SRT2104. (**D**) Cluster analysis heatmap of the 44 differentially abundant proteins in TA muscle between vehicle- and SRT2104-treated *mdx* mice. (**E**) Pie charts of GO terms enrichment analyses in TA muscle showing differentially express protein enrichments relative to Biological processes.

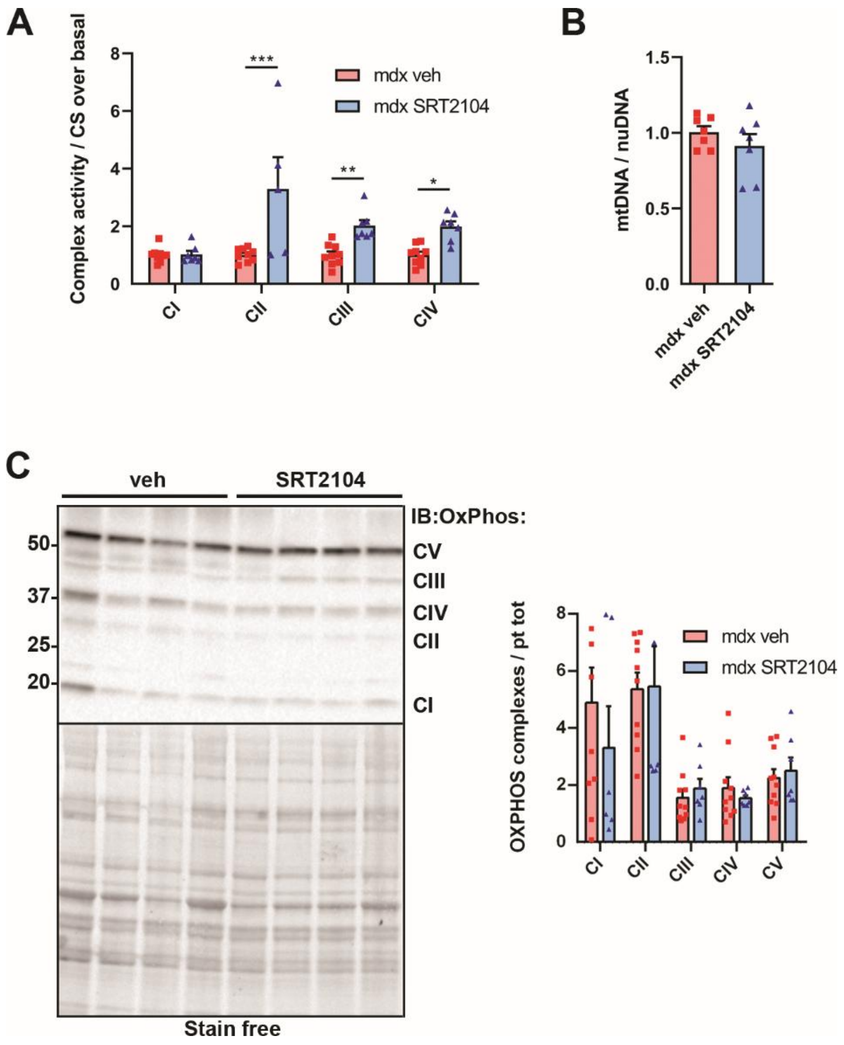

Figure S6. SRT2104 boosted mitochondrial activity without affecting mitochondrial mass.

(**A**) Mitochondrial respiratory complexes activity analysis measured in muscle homogenates of GC from either vehicle- or SRT2104 treated m*dx* mice (*mdx* veh n=9; *mdx* SRT2104 n≥5). (**B**) mtDNA quantification through qPCR in TA from either vehicle- or SRT2104-treated *mdx* mice (*mdx* veh n=7; *mdx* SRT2104 n=7). (**C**) Representative immunoblot for mitochondrial OxPhos complexes in GC muscle protein extracts of *mdx* mice treated or not with SRT2104 (CI-IV: Complex I-IV). Stain-free total protein content is provided below. Densitometric quantifications for the different mitochondrial complexes normalized on stain-free total protein content are provided (right panel) (*mdx* veh n=10; *mdx* SRT2104 n≥6). Molecular weights (kDa) are indicated on the left.

Table 1: List of primers used for RTqPCR (for mtDNA and ChIP analyses) and RTqPCR (F: forward; R: reverse).

	Gene accession number	Primer sequence	Amplicon length
**RNAse P**	NC_000085	F: 5'-GAAGGCTCTGCGCGGACTCG-3'<br>R: 5'-CGAGAGACCGGAATGGGGCCT-3'	119 bp
**CytB**	NC_005089.1	F: 5'- ACGCCATTCTACGCTCAATC -3'<br>R: 5'- GCTTCGTTGCTTTGAGGTAT-3'	110 bp
**RNAse P**	NC_000085	F: 5'-GAAGGCTCTGCGCGGACTCG-3'<br>R: 5'-CGAGAGACCGGAATGGGGCCT-3'	119 bp
**36B4**	NM_007475	F: 5'-AGGATATGGGATTCGGTCTCTTC-3'<br>R: 5'-TCATCCTGCTTAAGTGAACAAACT-3'	143 bp
**COL1A1**	NM_007742.4	F: 5'-GCTCCTCTTAGGGGCCACT-3'<br>R: 5'-CCACGTCTCACCATTGGGG-3'	103 bp
**CTGF**	NM_010217.2	F: 5'-GGGCCTCTTCTGCGATTTC-3'<br>R: 5'-ATCCAGGCAAGTGCATTGGTA-3'	151 bp
**α-SMA**	NM_007392.3	F: 5'-GTCCCAGACATCAGGGAGTAA-3'<br>R: 5'-TCGGATACTTCAGCGTCAGGA-3'	102 bp
**TGF-β**	NM_011577	F: 5'-AAACGGAAGCGCATCGAA -3'<br>R: 5'-GGGACTGGCGAGCCTTAGTT-3'	63 bp
**KLF4**	NM_010637.3	F: 5'-CAGTGGTAAGGTTTCTCGCC-3'<br>R: 5'-GCCACCCACACTTGTGACTA-3'	104 bp
**CEBPA**	NM_001287514	F: 5'-TATGACATCAGCGCCTACATCGA-3'<br>R: 5'-GTCGGCTGTGCTGGAAGAG-3'	76 bp
**CEBPB**	NM_001287738.1	F: 5'-GCCAAGAAGACGGTGGACAA -3'<br>R: 5'-ACAAGTTCCGCAGGGTGCT-3'	205 bp
**MED23**	NM_001166416.2	F: 5'-TCGGAAAATCATTGGAGGAG-3'<br>R: 5'-CAATAGGCAGGCATTTCGTT-3'	169 bp
**p21**	NM_001111099.2	F: 5'- GCAGAATAAAAGGTGCCACAG-3'<br>R: 5'- CGTCTCCGTGACGAAGTCAA-3'	194 bp

Table 2: List of antibodies used, and dilution applied in immunofluorescence (IF) and western blot (WB).

Epitope	Product name (catalogue number)	Manufacturer	Species	Dilution
Myosin Heavy Chain (MyHC)	MF-20	Developmental Studies Hybridoma Bank	Mouse	1:50 IF
Laminin A	L9393	Sigma-Aldrich	Rabbit	1:100 IF
MyHC-emb	sc-53091	Santa Cruz Biotechnology	Mouse	1:100 IF
CD45	CD45-FITC (130-102-491)	Miltenyi-Biotec	Rat	1:100 IF
OXPhos	OXPhos Rodent WB antibody cocktail (45-8099)	Invitrogen	Mouse	1:500 WB
Acetylated-Lysine	Acetylated-Lysine Antibody (9441)	Cell Signaling	Rabbit	1:1000 WB
Myozein1	Anti MYOZ-1 (HPA038437)	Sigma-Aldrich	Rabbit	1:100 IF
Actin	Alexa Fluor 546 Phalloidin (A22283)	Thermo Fisher Scientific		1:50 IF
Mouse-IgG (H+L)	Goat anti-Mouse IgG, Alexa Fluor™ 546 A-11030	Thermo Fisher Scientific	Goat	1:100 IF
MyoG	F5D	Developmental Studies Hybridoma Bank	Mouse	1:1000 WB
MyoD (5.8A)	sc-32758	Santa Cruz Biotechnology	Mouse	1:1000 WB
SIRT1 (SIR11)	S5196	Sigma-Aldrich	Mouse	1:1000 WB

Table 3: List of the differentially acetylated lysine sites in TA muscles**.**

Accession	GeneName	Kposition	Protein name	Acetyl (K) Probabilities	PEP	Score	P.Value	Status in SRT2104 group
P05977	MYL1	K156	Myosin light chain 1/3, skeletal muscle isoform	HVLATLGEK(*)MKEEEVEALLAGQEDSNGCINYEAFVK	0,003683	54,703	0,002299	Up
P07310	CKM	K223	Creatine kinase M-type	GIWHNDNK(*)SFLVWVNEEDHLR	0,002107	64,265	0,005336	Up
P07310	CKM	K196	Creatine kinase M-type	SMTEQEQQQLIDDHFLFDK(*)PVSPLLLASGMAR	4,04E-19	114,63	0,023577	Down
P07310	CKM	K172	Creatine kinase M-type	GK(*)YYPLKSMTEQEQQQLIDDHFLFDKPVSPLLLASGMAR	5,32E-08	81,732	0,000215	Down
A2A6J0	TNNT3	K52	Troponin T, fast skeletal muscle	QNK(*)DLMELQALIDSHFEAR	0,001749	66,827	0,010898	Down
A8Y5P4	MAP7D1	K270	MAP7 domain containing 1	KTSGSRCSVSAVNLPKHVDSIINK(*)R	0,075252	41,76	0,01854	Down
Q5SX40	MYH2	K1707	Myosin-1 (Myosin heavy chain 1) (Myosin heavy chain 2x)	K(*)IAEQELLDASERVQLLHTQNTSLINTK	6,02E-06	78,45	0,002803	Up
P06151	LDHA	K5	L-lactate dehydrogenase A chain (LDH-A)	ATLK(*)DQLIVNLLKEEQAPQNK	3,68E-129	329,21	0,039348	Up
P09411	PGK1	K91	Phosphoglycerate kinase 1	SLLGK(*)DVLFLKDCVGPEVENACANPAAGTVILLENLR	2,83E-08	94,614	0,031597	Up
Q5SX39	MYH8	K1507	Myosin-4 (Myosin heavy chain 2b) (MyHC-2b) (Myosin heavy chain 4)	ENK(*)NLQQEISDLTEQIAEGGKHIHELEK	4,25E-06	77,522	0,011736	Up
P48962	SLC25A4	K23	ADP/ATP translocase 1 (ADP,ATP carrier protein 1)	GDQALSFLKDFLAGGIAAAVSK(*)TAVAPIER	3,47E-28	152,49	0,000798	Down
P52480-2	PKM	K337	Pyruvate kinase PKM	K(*)PRPTRAEGSDVANAVLDGADCIMLSGETAKGDYPLEAVR	0,00011	51,216	0,005028	Down
P97457	MYLPF	K63	Myosin regulatory light chain 11	LNVK(*)NEELDAMMK(*)EASGPINFTVFLTMFGEK	4,81E-06	74,474	0,004864	Down
P97457	MYLPF	K72	Myosin regulatory light chain 11	NEELDAMMK(*)EASGPINFTVFLTMFGEK	1,94E-09	90,363	0,001052	Down
Q03265	ATP5A1	K506	ATP synthase subunit alpha, mitochondrial (ATP synthase F1 subunit alpha)	ITK(*)FENAFLSHVISQHQSLLGNIR	5,55E-16	144,77	0,021104	Up
Q5SX39	MYH4	K1655	Myosin-4 (Myosin heavy chain 2b) (MyHC-2b) (Myosin heavy chain 4)	NTQGMLK(*)DTQLHLDDALRGQDDLKEQLAMVERR	0,008278	47,006	0,000367	Down
Q5SX39	MYH4	K601	Myosin-4 (Myosin heavy chain 2b) (MyHC-2b) (Myosin heavy chain 4)	AEAHFSLVHYAGTVDYNIIGWLDKNK(*)DPLNETVVGLYQK	6,13E-05	63,761	0,000473	Down
Q5SX39	MYH4	K599	Myosin-4 (Myosin heavy chain 2b) (MyHC-2b) (Myosin heavy chain 4)	AEAHFSLVHYAGTVDYNIIGWLDK(*)NKDPLNETVVGLYQK	6,13E-05	63,761	0,02754	Down
Q5SX39	MYH4	K888	Myosin-4 (Myosin heavy chain 2b) (MyHC-2b) (Myosin heavy chain 4)	MVALMQEK(*)NDLQLQVQAEADGLADAEERCDQLIK	7,44E-05	70,268	0,031232	Up
Q5SX39	MYH4	K1461	Myosin-4 (Myosin heavy chain 2b) (MyHC-2b) (Myosin heavy chain 4)	NFDKVLAEWK(*)QKYEETQAELEASQKESR	8,62E-06	76,765	0,001082	Down
Q5SX40	MYH1	K1538	Myosin-1 (Myosin heavy chain 1) (Myosin heavy chain 2x)	K(*)QIEQEKSELQAALEEAEASLEHEEGKILR	8,35E-09	84,515	0,009477	Up
Q5SX40	MYH1	K891	Myosin-1 (Myosin heavy chain 1) (Myosin heavy chain 2x)	MVALMQEK(*)NDLQLQVQSEADSLADAEERCDQLIK	4,60E-05	72,292	0,000259	Down
Q7TQ48-1	SRL	K477	Sarcalumenin	SHIDK(*)TLMLNEDKPADDYSAVLQR	0,031725	47,728	0,019816	Down
Q8CGP2	HIST1H2BP	K109	Histone H2B type 1-P	LLLPGELAK(*)HAVSEGTK	0,051378	67,605	0,007462	Down
Q99LX0	PARK7	K99	Parkinson disease protein 7 homolog	K(*)GLIAAICAGPTALLAHEVGFGCK	7,80E-05	86,551	5,14E-05	Up
Q9CPP6	NDUFA5	K67	NADH dehydrogenase [ubiquinone] 1 alpha subcomplex subunit 5	K(*)LEALLQGGEVEEVILQAEKELSLAR	1,48E-06	93,397	0,001433	Down
Q9CPQ9	ALDOART1	K87	Fructose-bisphosphate aldolase	QLLLTADDRVNPCIGGVILFHETLYEK(*)ADDGRPFPQVIK	2,56E-07	81,204	0,000132	Down
P05064	ALDOART1	K108	Fructose-bisphosphate aldolase A	SKGGVVGIK(*)VDKGVVPLAGTNGETTTQGLDGLSER	6,87E-06	80,652	0,000933	Down
