# Supplementary figures and images for: The SIRT1 activator SRT2104 exerts exercise mimetic effects and promotes Duchenne muscular dystrophy recovery
Source: Cell Death Dis. 2025 Apr 7;16(1):259. doi: 10.1038/s41419-025-07595-z (PMC11977210; doi:10.1038/s41419-025-07595-z)

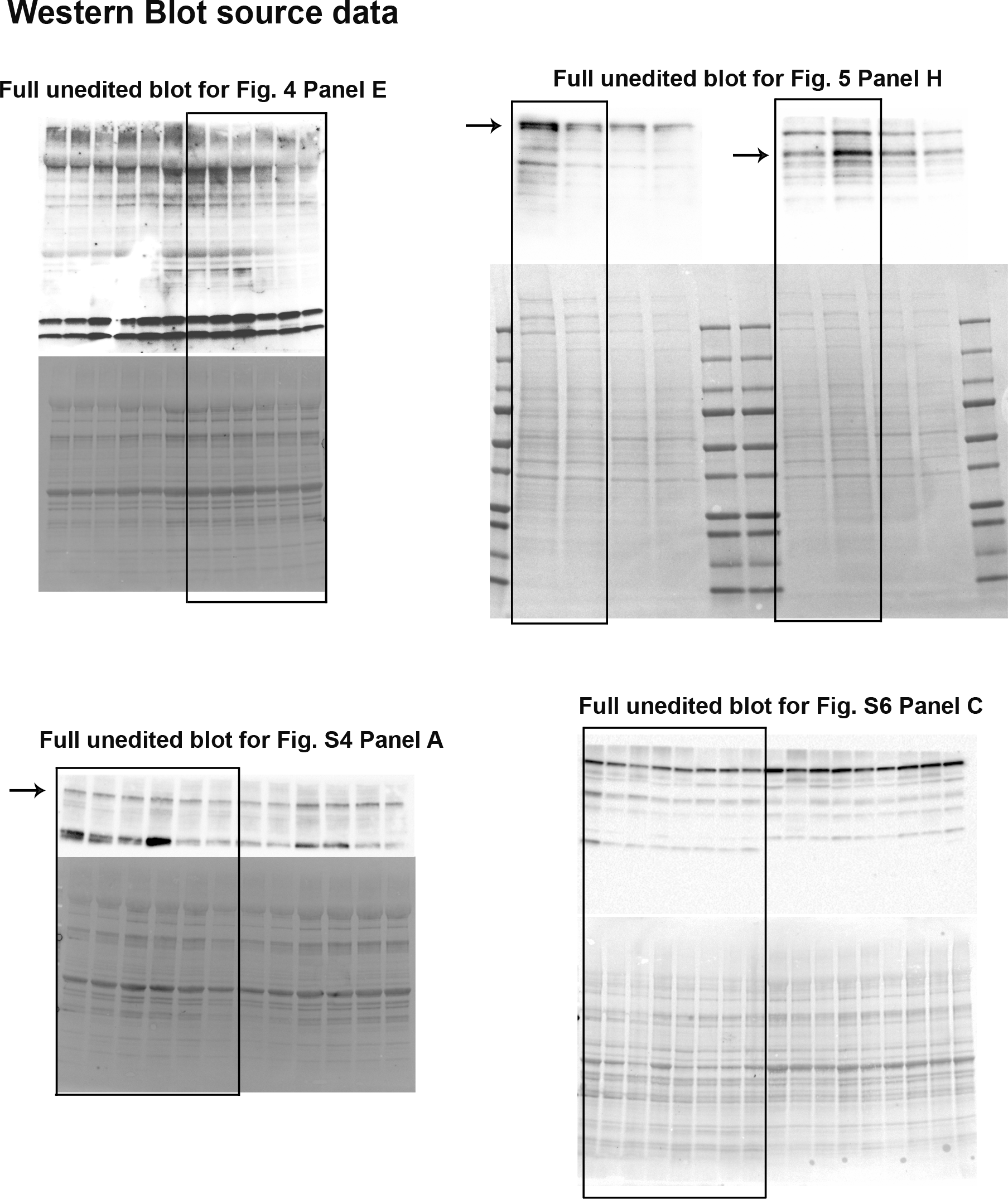

Supplement: Supplementary file 2 — uncropped original western blots [file 41419_2025_7595_MOESM2_ESM.tif]
